# Flash nanoprecipitation allows easy fabrication of pH-responsive acetalated dextran nanoparticles for intracellular release of payloads

**DOI:** 10.1186/s11671-023-03947-w

**Published:** 2024-01-04

**Authors:** Krystal A. Hughes, Bishal Misra, Maryam Maghareh, Parinya Samart, Ethan Nguyen, Salik Hussain, Werner J. Geldenhuys, Sharan Bobbala

**Affiliations:** 1 Department of Pharmaceutical Sciences, West Virginia University School of Pharmacy, Morgantown, WV 26505 USA; 2 Department of Clinical Pharmacy, West Virginia University School of Pharmacy, Morgantown, WV 26505 USA; 3 Siriraj Center of Excellence for Stem Cell Research, Faculty of Medicine Siriraj Hospital, Mahidol University, Bangkok, Thailand; 4 Department of Microbiology, Immunology and Cell Biology, West Virginia University School of Medicine, Morgantown, WV 26505 USA; 5 Department of Physiology, Pharmacology and Toxicology, West Virginia University, Morgantown, WV 26505 USA; 6 Department of Neuroscience, West Virginia University School of Medicine, Morgantown, WV 26505 USA

**Keywords:** Flash nanoprecipitation, Nanoparticles, Intracellular release, Dextran, pH-responsive, Surfactants

## Abstract

Acetalated dextran (Ac-Dex) nanoparticles are currently of immense interest due to their sharp pH-responsive nature and high biodegradability. Ac-Dex nanoparticles are often formulated through single- or double-emulsion methods utilizing polyvinyl alcohol as the stabilizer. The emulsion methods utilize toxic organic solvents such as dichloromethane or chloroform and require multi-step processing to form stable Ac-Dex nanoparticles. Here, we introduce a simple flash nanoprecipitation (FNP) approach that utilizes a confined impinging jet mixer and a non-toxic solvent, ethanol, to form Ac-Dex nanoparticles rapidly. Ac-Dex nanoparticles were stabilized using nonionic PEGylated surfactants, D-α-Tocopherol polyethylene glycol succinate (TPGS), or Pluronic (F-127). Ac-Dex nanoparticles formed using FNP were highly monodisperse and stably encapsulated a wide range of payloads, including hydrophobic, hydrophilic, and macromolecules. When lyophilized, Ac-Dex TPGS nanoparticles remained stable for at least one year with greater than 80% payload retention. Ac-Dex nanoparticles were non-toxic to cells and achieved intracellular release of payloads into the cytoplasm. In vivo studies demonstrated a predominant biodistribution of Ac-Dex TPGS nanoparticles in the liver, lungs, and spleen after intravenous administration. Taken together, the FNP technique allows easy fabrication and loading of Ac-Dex nanoparticles that can precisely release payloads into intracellular environments for diverse therapeutic applications.

**Graphical abstract:**

pH-responsive Acetalateddextran can be formulated using nonionic surfactants, such as TPGS or F-127, for intracellular release of payloads. Highly monodisperse and stable nanoparticles can be created through the simple, scalable flash nanoprecipitation technique, which utilizes a confined impingement jet mixer.
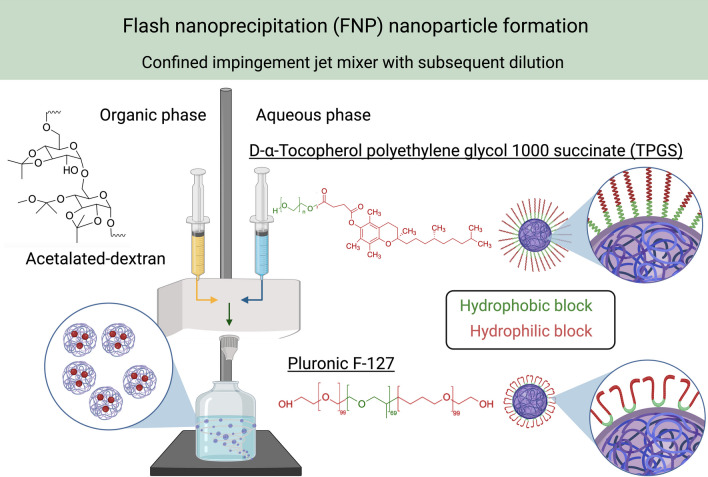

**Supplementary Information:**

The online version contains supplementary material available at 10.1186/s11671-023-03947-w.

## Introduction

Nanoparticle-based drug delivery platforms have become increasingly prevalent in pharmaceutical research. Numerous FDA-approved therapeutics that utilize nanoparticles have impacted clinical care for various disease states [1]. Effective intracellular drug delivery, particularly subcellular organelle delivery, is highly sought for enhanced therapeutic efficacy and decreased systemic side effects [2, 3]. Stimulatory-responsive nanoparticles have been utilized to achieve endosomal/lysosomal escape or cytoplasmic release of therapeutics [2, 4]. Stimulatory-responsive nanoparticles that can release their encapsulated cargo when exposed to specific biological stimuli such as pH, temperature, and redox conditions are of great interest for drug and vaccine delivery applications [5]. Most commonly, stimulatory-responsive nanoparticles utilize pH-triggered release mechanisms [2]. This is partly due to the heterogeneity of environmental pH in intracellular and extracellular spaces [6]. Notably, intracellular pH is ideal for exploiting stimuli-responsive nanoparticles [7]. Nanoparticles witness an extracellular physiological pH (pH 7.4) before endocytosis to intracellular endosomal pH conditions (pH 5.5–6) to the more acidic lysosome (pH 4) [6]. The pH-responsive nanoparticle will undergo physiochemical alterations upon exposure to these acidic environments, causing nanoparticle payload release. pH-responsive nanoparticles may either be naturally or synthetically derived. Natural polymers with pH responsiveness include hyaluronic acid, alginic acid, heparin, chitosan, carboxymethyl cellulose, and dextran [8]. Synthetic polymers that can possess pH responsiveness include polypeptide derivatives, including poly(L-glutamic acid) (PGA), poly(histidine) (PHIS), and poly(aspartic acid) (PASA) [8]. These polymers have been widely studied for various biomedical applications.

Acetalated dextran (Ac-Dex) polymer, which is synthesized from natural carbohydrate dextran, is currently of tremendous interest due to its biocompatible and biodegradable nature and for precision intracellular release of therapeutics [9]. Introducing the acetal group makes dextran not only pH-responsive but also hydrophobic, which allows the fabrication of nanoparticles [10, 11]. In acidic environments, the acetal group is hydrolyzed, resulting in the hydrophobic Ac-Dex converting back to the hydrophilic dextran, leading to the collapse of nanoparticles and the escape of therapeutics [10]. Studies have demonstrated that Ac-Dex can deliver numerous therapeutics, including small molecules [12, 13], gene therapy [14, 15], and adjuvants for vaccine delivery [16, 17].

Ac-Dex nanoparticles are often fabricated through conventional single- or double-emulsification techniques [10–12]. The formulation process of emulsification has numerous drawbacks; it is an exothermic process that can produce less stable and polydisperse nanoparticles with poor batch-to-batch variations [18]. Efforts have been made to utilize other techniques to form Ac-Dex nanoparticles outside of single and double emulsions, including conventional nanoprecipitation, microfluidic nanoprecipitation, spray drying, and electrospray [19]. However, some of the major limitations of these techniques include using toxic organic solvents, requiring longer drying times to remove non-volatile organic solvents, and employing elevated temperatures that do not suit the encapsulation of thermolabile compounds.

Flash nanoprecipitation (FNP) is a rapid, turbulent mixing procedure for the scalable formation of nanoparticles [20]. FNP utilizes either multi-inlet vortex (MIV) or confined impingement (CIJ) mixers. CIJ is a simple, two-stream mixer that encapsulates hydrophobic and hydrophilic payloads into nanoparticles. In the FNP process, an organic solvent containing a hydrophobic polymer or payloads and a miscible antisolvent aqueous phase containing a surfactant or hydrophilic payloads simultaneously impinge in a mixer [21, 22]. This rapid mixing process induces supersaturation conditions, leading to the precipitation of hydrophobic components [21, 22]. FNP has been widely studied for self-assembling morphologically diverse nanoparticles or stabilizing hydrophobic small molecule drugs using amphiphilic block copolymers [23]. Recent findings show that FNP could stabilize hydrophobic polymer cores and inorganic colloids [24]. However, these findings were confined to hydrophobic homopolymers such as polystyrene (PS) and polylactide (PLA) and utilized only tetrahydrofuran (THF) as a solvent [25, 26]. It is important to explore rapid fabrication approaches to stabilize novel polymers that are otherwise hard to formulate with enhanced stability using simple, non-toxic organic solvents.

In this study, we investigated the role of FNP in stabilizing the hydrophobic Ac-Dex polymer core using PEGylated non-ionic surfactants, D-α-Tocopherol polyethylene glycol succinate (TPGS) and Pluronic (F-127). A recent study has shown that Ac-Dex can be successfully stabilized with F-127 through a nanoprecipitation reaction [27]. However, this does not utilize a CIJ mixer, which has the advantage of ease and increased scalability. Additionally, acetone was utilized as the organic solvent. Commonly utilized solvents in FNP include acetonitrile, chloroform, methanol, and THF, which are Class 2 solvents as classified by the FDA and US Pharmacopeia pharmaceutical industry guidance documents. In contrast, ethanol is classified as a Class 3 solvent [28]. Class 3 solvents are considered less toxic and not a human health hazard [28]. Another study has explored Ac-Dex using ethanol before, but this has been seen with the electrospray formulation method, which has been shown to make micrometer-size particles [29]. Other short-chain alcohols have also been used in Ac-Dex formulation, as seen with isopropyl alcohol, but this method also explored the electrospray formulation method [30].

Here, we utilized ethanol as an organic solvent to dissolve Ac-Dex polymer and an aqueous phase containing surfactants. Of note, TPGS and F-127 have been approved by the FDA as excipients for their favorable safety profiles [29]. The role of TPGS or F-127 in stabilizing Ac-Dex is unknown, and therefore, we optimized the surfactant concentration to stabilize Ac-Dex nanoparticles. Next, Ac-Dex nanoparticles were examined for their ability to load diverse payloads, and for their stability over time. Furthermore, toxicity, cellular uptake and release of payloads and in vivo biodistribution of nanoparticles were explored. The formulation approach introduced here will be an easy way to manufacture Ac-Dex nanoparticles that can address intracellular delivery challenges associated with therapeutics for numerous biomedical applications. This pH-responsive polymeric nanoparticle and formulation technique has numerous applications that would be worthwhile exploring due to the ease and scalability of fabrication. Multiple diseases including cancer and infectious diseases caused by intracellular pathogens often require drugs to be delivered at higher concentrations inside the diseased cells with greater precision and intracellular delivery of therapeutics via pH-responsive nanoparticles will be of huge benefit [2]. Nanoparticles that are pH-responsive can also play a vital role in prophylactic and therapeutic vaccine delivery applications [31, 32]. For example, immunological adjuvants and nucleic acid vaccines need to be delivered to different intracellular locations of antigen-presenting cells such as endosomes and cytoplasm to generate potent immune responses against antigens. Of note, utilizing pH-responsive nanoparticles in vaccine strategies may effectively stimulate immune cells with low doses of vaccine components.

## Materials and methods

### Materials

Dextran from *Leuconostoc mesenteroides* (MW 9,000–11,000), 2-methoxypropene, D-α-Tocopherol polyethylene glycol 1000 succinate, Pluronic™ F-127, albumin-fluorescein isothiocyanate conjugate protein bovine, rhodamine B, and D-mannitol 97 + %, were purchased through Sigma-Aldrich (Burlington, MA). DiD perchlorate was obtained from MedChemExpress (Monmouth Junction, NJ). Doxorubicin hydrochloride and curcumin (Natural), D-( +)-trehalose dihydrate, and D-( +)-Sucrose, were purchased through TCI America (Portland, OR). Calcein was purchased through MP Biomedicals (Solon, OH), and chlorpromazine (hydrochloride) and genistein was purchased through Cayman Chemical (Ann Arbor, MI). Thiazolyl blue tetrazolium bromide 98% was purchased through Alfa Aesar (Haverhill, MA). The CCK8 Cell Counting Kit was purchased through Dojindo Molecular Technologies (Gaithersburg, MD). The Zombie Aqua stain fixable viability kit was obtained from BioLegend (San Diego, California). NucBlue™ Live ReadyProbes™ Reagent and SnakeSkin™ Dialysis Tubing (10 K MWCO) were purchased through Thermo Fisher Scientific (Waltham,MA). The confined impingement jet mixer was obtained from Holland Applied Technologies (Burr Ridge, IL), which fabricates these mixers in collaboration with the Department of Chemical and Biological Engineering at Princeton University. The nozzles used in the FNP process were Threaded Luer Adapter, 0.050" Bore, Female Luer 1/4-28 Flat Bottom, and the bottom nozzle and tubing, which were an Idex Flangeless Fitting, Standard Knurl, 1/16" OD Tubing, 1/4-28 Flat-Bottom, and Idex Chromatography Tubing, 1/32" OD × 0.020" were purchased from Cole-Parmer (Vernon Hills, IL). The syringes used in the formulation process were sterile Luer Slip 1 mL plastic NORM-JECT© syringes purchased from Grainger (Lake Forest, IL).

### Acetalated dextran synthesis

Ac-Dex polymer was synthesized by introducing a pH-responsive acetal group as described previously [1] Briefly, 1 g of dextran was weighed, transferred into a Schlenk flask, purged with nitrogen, and added 10 mL of anhydrous dimethylsulfoxide (DMSO). Following complete solubilization of dextran, 15.6 mg of pyridinium p-toluenesulfonate (0.062 mmol) and 2-methoxy propene (3.4 mL, 37 mmol) were added to the flask, sealed, and placed under nitrogen gas. After 3 h, the reaction was quenched using triethylamine (1 mL, 7 mmol) for 5 min and the resulting mixture was precipitated in 150 mL of distilled water (pH 9). The precipitate was centrifuged at 1000 × g for 10 min and the pellet was washed twice with water (pH 9) before lyophilizing to remove residual water and collect the Ac-Dex powder. The reaction yield was > 95%, and the cyclic acetal coverage of Ac-Dex was determined through ^1^H-NMR using deuterium chloride (DCl) and deuterium oxide (D_2_O) as solvents [33], which confirmed 63.1% of cyclic acetal coverage.

### Flash nanoprecipitation preparations of nanoparticles

The nanoparticle formulations were prepared with a confined impingement jet mixer (CIJ). The Ac-Dex was weighed to 20 mg and dissolved in 500 μl of ethanol. Any hydrophobic compounds were weighed separately and dissolved in the same ethanol solution containing the polymer. For the surfactant, either D-α-tocopherol polyethylene glycol 1000 succinate (TPGS) or Pluronic (F-127) were weighed to 5 mg and then dissolved with 500 μl of deionized water. Any hydrophilic compounds were weighed separately and dissolved in the same water solution containing the surfactant. The two syringes for impingement contained the organic phase and the antisolvent, aqueous phase. The two syringes were rapidly impinged via hand-operated impingement simultaneously through the CIJ mixer at a rate of 1 mL s^−1^ and collected in a 10 mL vial containing 2 mL of deionized water for rapid quenching of the nanoparticle formation process. The organic solvent was removed overnight through evaporation. The samples were then centrifuged at 5000xg for 5 min. The supernatant was discarded to remove unencapsulated materials, excess surfactant, and residual organic solvent. The nanoparticle pellet was washed twice by resuspending in deionized water and centrifugation. After washing, the pellet was finally resuspended in phosphate-buffered saline (PBS). Samples were then stored at 4 °C for further usage. For lyophilization studies, samples were added with cryoprotectants, kept overnight at − 20 °C, and lyophilized.

### Formulation optimization studies

Different percentages of surfactant TPGS, 2, 1, 0.5, and 0.1% w/v were utilized to form Ac-Dex nanoparticles. The 1% w/v surfactant that was optimized with Ac-Dex TPGS, was employed for Ac-Dex F-127 and analyzed. The organic phase was formulated using 500 μl of ethanol, DMSO, dimethylformamide (DMF), tetrahydrofuran (THF), and methanol. The formulations containing ethanol, methanol, and THF were spun overnight for evaporation, while the DMSO and DMF were dialyzed against deionized water. After dialysis or solvent evaporation, the nanoparticles were washed twice with deionized water and resuspended in PBS to a final volume of 2 mL before characterization.

### Preparation of Ac-Dex nanoparticles using O/W single-emulsion technique

The polymer Ac-Dex was weighed out to 20 mg and dissolved in 500 μL of chloroform to make the organic phase. Any hydrophobic compounds were weighed separately and dissolved in the chloroform solution containing the polymer. For stabilization, different concentrations of TPGS, F-127, or PVA (2.5% w/v) were prepared in water. The organic phase (500 μL) and an aqueous phase containing stabilizer (3 mL) were added to the 15 mL conical tube and then sonicated for 15 s at 70% amplitude to form an oil/water emulsion. The organic solvent chloroform was removed by stirring the emulsion overnight in the fume hood and nanoparticles were centrifuged and the pellet was washed twice with water to remove excess surfactant before suspending in PBS for further use.

### Size and zeta potential characterization

The size and zeta potential were measured by dynamic light scattering and electrophoretic light scattering, respectively, using the Malvern Zetasizer. For size and polydispersity index (PDI), 20 μl nanoparticle samples were diluted to 1 mL of PBS was placed into a cuvette before measuring the size and PDI. A capillary cuvette was used to measure zeta potential; this was also performed with 1 mL of PBS and 20 μl of the sample. All readings were taken in triplicate.

### Lyophilization of nanoparticle formulations

The cryoprotectants used during optimization included trehalose, mannitol, and sucrose. The cryoprotectants were added at 5–10% (w/v) to the samples and briefly vortexed. The samples were transferred to a 10 mL vial and placed at − 80 °C for 1 h. Once the samples were frozen, they were moved to the Lyovapor L-200 lyophilizer and processed overnight to collect the lyophilized cake.

### Stability determination

Nanoparticles were lyophilized with 10% (w/v) of mannitol as the cryoprotectant. After lyophilization, the nanoparticles were stored at 4 °C for a predetermined amount of time, either 1 day, 30 days, or one year. At each time point, lyophilized cakes were rehydrated with the same volume of PBS (as utilized initially) to form nanoparticles. The rehydrated nanoparticles were characterized for their physiochemical properties (size, PDI, and zeta potential), or retention of payloads, and the readings were taken in triplicate.

### Transmission electron microscopy (TEM)

Ac-Dex nanoparticles were characterized through transmission electron microscopy. Samples were applied to UV-treated, carbon-coated EM grids (Ted Pella, 01840-F) and stained immediately using 1% aqueous uranyl acetate. Micrographs were recorded on a JEOL 1010 microscope using an AMT XR611S-B CCD camera.

### Quantification of fluorophore encapsulation

DiD perchlorate (λEx = 644 nm; λEm = 665 nm), 12 μl (10 mg/ml) was added to the organic phase. Curcumin (λEx = 420 nm; λEm = 470 nm) was weighed to 1 mg and added to the organic phase. Rhodamine 6B (λEx = 546 nm; λEm = 568 nm) was weighed to 1 mg and added to the aqueous phase. Calcein AM (λEx = 495 nm; λEm = 515 nm) was weighed to 1 mg and added to the aqueous phase. Doxorubicin hydrochloride (λEx = 470 nm; λEm = 560 nm) was weighed to 1 mg and added to the aqueous phase. Fluorescein-labeled bovine serum albumin (FITC- BSA) (λEx = 495 nm; λEm = 515 nm) was weighed to 1 mg and added to the aqueous phase. All fluorescence readings were taken using a SpectraMax microplate reader. Encapsulation efficiency was determined by comparing the fluorescence before and after the samples were processed through centrifugation.

### Quantification of fluorophore retention in lyophilized formulations

Ac-Dex TPGS nanoparticles were loaded with DiD and analyzed before lyophilization. From a 2 mL of DiD formulation, 1 mL of the formulation was stored overnight at 4 °C, and the other 1 mL of the formulation was lyophilized with 10% (w/v) mannitol. The lyophilized cake was rehydrated to form nanoparticles and was centrifuged to remove the free drug and the pellet was resuspended in PBS and analyzed for DiD retention as compared to the pre-lyophilized sample stored at 4 °C.

### pH-responsive drug release studies

200 μl of Ac-Dex TPGS nanoparticles (10 mg/mL) were added to 2 mL microcentrifuge tubes with 500 μl of the appropriate buffer. Each acidic pH buffer was made by adding hydrochloric acid to PBS and monitored for the appropriate pH through a pH meter. Basic pH buffers were made by adding sodium hydroxide to phosphate buffer saline (PBS) and monitored for the appropriate pH through a pH meter. The microcentrifuge tubes were placed on an orbital incubator shaker (Benchmark Scientific Incu-Shaker Mini) for various time points (n = 3 per buffer condition) at 37 °C. After incubation, the microcentrifuge tubes were centrifuged at 5000xG for 5 min. 150 μl of the supernatant was collected into 96 black well-clear bottom polystyrene plates (n = 3 per sample). The fluorescence for each sample was measured using a SpectraMax microplate reader.

### Serum stability studies

200 μl of Ac-Dex TPGS nanoparticles (10 mg/mL) loaded with DiD were added to 2 mL microcentrifuge tubes with 500 μl of 10% fetal bovine serum (FBS). The microcentrifuge tubes were placed on an orbital incubator shaker (Benchmark Scientific Incu-Shaker Mini) for various time points (n = 3 per condition) at 37 °C. After incubation, the microcentrifuge tubes were centrifuged at 5000xG for 5 min. 50 μl of the supernatant was collected into 96 black well-clear bottom polystyrene plates (n = 3 per sample). The fluorescence for each sample was measured using a SpectraMax microplate reader. The Ac-Dex TPGS nanoparticles then underwent size characterization after the last time point.

### Cell culture

Raw 264.7 macrophages (murine macrophage cell line) were obtained from Invivogen (San Diego, CA) and were cultured with Dulbecco’s Modified Eagle Medium (DMEM) supplemented with 10% FBS and 1% penicillin/streptomycin antibiotics. The cells were passaged by mechanical scraping after reaching 70–80% confluency in a T75 polystyrene tissue culture-treated flask. All cells were cultured at 37 °C, 5% CO2.

MDA-MB-231 triple-negative breast cancer GFP-expressing cells were obtained from Dr. Paul Lockman's Lab at West Virginia University and were cultured with Roswell Park Memorial Institute 1640 (RPMI) supplemented with 10% FBS and 1% penicillin/streptomycin antibiotics. The cells were passaged through trypsinization after reaching 70–80% confluency in a T75 polystyrene tissue culture-treated flask. The cells were centrifuged at 800 RCF for 5 min and then resuspended with new media before they were plated again. All cells were cultured at 37 °C, 5% CO2.

REH-CRL-8286 B-cell acute lymphocytic leukemia cells were obtained from Dr. Werner Geldenhuyes's lab at West Virginia University and were cultured with Roswell Park Memorial Institute 1640 (RPMI) supplemented with 10% FBS and 1% penicillin/streptomycin antibiotics. Cells were plated in either T25 or T75 non-treated tissue culture flasks. The cells were fed with additional media every three days. All cells were cultured at 37 °C, 5% CO2.

### Flow cytometric analysis of nanoparticle uptake and determination of uptake mechanism

For each specified cell line, 200,000 cells were seeded per well in 6 well polystyrene plates, or 100,000 cells were seeded per well in 12 well polystyrene plates. Adherent cells were allowed to adhere overnight at 37 °C, 5.0% CO2. Cells were treated with 20 μl of nanoparticle formulations for 15 min, 30 min, 1 h, 2 h, 6 h, and 24 h and incubated at 37 °C, 5.0% CO2. After the nanoparticle treatment period, cells were harvested by either mechanical scraping (macrophages), trypsinization (MDA-231), or aspiration (REH). Zombie Aqua viability dye was used to stain cells for 30 min at 4 °C to assess cytotoxicity. Each sample had 30,000 single-cell events. To determine a mechanism of endocytosis, cells were treated with chlorpromazine (CPZ, 15 μM), a clathrin-mediated endocytosis inhibitor, or genistein 200 μM for 6 h, a caveolae-mediated endocytosis inhibitor, before incubating nanoparticles. A BD LSRFortessa 4-Laser Flow Cytometer with an FSC PMT Detector and high throughput system was used to perform the flow cytometry studies, and Cytobank was used for data analysis. All samples were examined as triplicates, and the percentage of the live cell population that demonstrated DiD uptake was quantified.

### MTT assay

Cell viability was assessed for Raw 264.7 macrophages and MDA-MB-231 through MTT (3-(4,5-dimethylthiazolyl-2)-2,5-diphenyltetrazolium bromide) assays. The cells were cultured at a density of 3 × 10^4^ cells per well (n = 4 per condition), and tissue culture-treated 96 well plates were used. Adherent cells were allowed to adhere overnight at 37 °C, 5.0% CO2. The tested conditions were performed on cells treated with 10 μl nanoparticles for 24 to 48 h at 37 °C, 5.0% CO2. Cells were treated with 0.5 mg/mL thiazolyl blue tetrazolium bromide (MTT) and incubated at 37 °C, 5.0% CO2 for 4 h. The media in each well was aspirated, and 150 μl of Dimethylsulfoxide (DMSO) was added to each well to dissolve the formazan crystals. The viability was quantified with a SpectraMax microplate reader, which was used to measure the absorbance at 570 nm. Viability was determined as a percentage and was calculated by using the mean absorbance of the experimental groups and dividing that by the mean absorbance of PBS-treated cells.

### CCK-8 assay

Cell viability was assessed for REH-CRL-8286 through Cell Counting Kit-8 (CCK-8) assays (Dojino). The cells were cultured at a density of 1 × 10^4^ cells per well (n = 4 per condition), and non-tissue culture-treated 96 well plates were used. The conditions were tested on cells treated with 10 μl nanoparticles for 24, 48, or 72 h at 37 °C, 5.0% CO2. After the designated treatment time, 10 μl of the WST-8 formazan dye solution was added to each well for 4 h. The viability was quantified with a SpectraMax microplate reader, which was used to measure the absorbance at 450 nm. The absorbance was also taken at 600 nm and subtracted from the absorbance recorded at 450 nm to account for turbidity within the cell suspension. Viability was determined as a percentage and was calculated by using the mean absorbance of the experimental groups and dividing that by the mean absorbance of PBS-treated cells.

### Confocal determination of intracellular fluorophore release

The cytosolic release was analyzed using a Nikon A1R confocal fluorescence microscope (60X oil lens) equipped with three laser lines 405 nm (NucBlue LiveReady Probe) (Invitrogen), and 640 nm (DiD dye) and 550 nm (Rhodamine B). The brightfield (transmitted light) was also imaged. Galvano scanning, 1.2 pinhole, with 1/8th frame/sec and a 512 size, was used while imaging. Raw 264.7 macrophages were seeded at a density of 2 × 10^4^ cells per well into 8-well chambered slides. Adherent cells were allowed to adhere overnight at 37 °C, 5.0% CO2. 20 μl of the nanoparticles were added the next day and left to incubate for 24 h. The media was then aspirated, and the wells were washed three times with PBS. NucBlue was added directly before imaging, and one drop was added per well. For REH-CRL-8286, cells were first seeded at a density of 5 × 10^4^ cells per well, using 12 well non-treated tissue culture plates. 20 μl of the nanoparticles was added to each well, and the plates were left overnight to incubate at 37 °C, 5.0% CO2. Following incubation, the wells were aspirated, and the cell suspension was centrifuged at 400 RPM for 5 min. The supernatant of dead cells and non-endocytosed nanoparticles was aspirated, and the cell pellet was resuspended with RPMI. 300 μl of the cell suspension per well was then plated into Poly-D-Lysine coated 8-well chambered slides. The cells adhered overnight and incubated at 37 °C, 5.0% CO2. The media was then aspirated, and the wells were washed once with phosphate-buffered saline (PBS), and one drop per well of NucBlue was added directly before imaging. Confocal images were subsequently processed using the ImageJ software (Version 2.1.0/1.53c).

### Poly-D-Lysine coated 8-well chambered slides

300 μl of Poly-D-Lysine (PDL) (Gibco) was added to each of the 8-well chambered slides and left out to incubate at room temperature for 1 h. The PDL was then aspirated, and the wells were washed three times with PBS. After the last wash, the remaining PBS was aspirated, and the coated chamber slide was left to dry overnight. When the coated plates were not immediately used, they were tightly wrapped with a Parafilm™.

### Animals

Animal studies were approved by the West Virginia University Institution of Animal Care and Use Committee. All methods were carried out in accordance with relevant institutional guidelines and regulations. 4–6 weeks old male C57BL/6 J mice were maintained in the animal facility.

### IVIS imaging

C57BL/6 J male mice were intravenously injected through the retroorbital plexus either with 100 µL of indocyanine green (ICG)-loaded Ac-Dex nanoparticles or 100 µL PBS control. After 4 or 24 h, the mice were sacrificed and perfused. The liver, kidney, lungs, heart, spleen, brain, and spleens were harvested for the IVIS imaging using the IVIS SpectrumCT (PerkinElmer). The total radiant efficiency ((p/s)/(µW/cm^2^)) of ICG was reported to measure the biodistribution of the Ac-Dex TPGS nanoparticles.

### Statistical analysis

Statistical analyses were performed using GraphPad Prism software (version 9.5.0). All experiments were completed with a minimum of 3 replicates. One-way ANOVAs were utilized, followed by Tukey’s multiple comparison test; ordinary unpaired T-tests were also utilized. Significance was determined with differences with a *p*-value < 0.5, and values are reported as mean ± standard deviation.

## Results and discussion

### Flash nanoprecipitation allows rapid fabrication and loading of Ac-Dex nanoparticles.

**Fig. 1:**
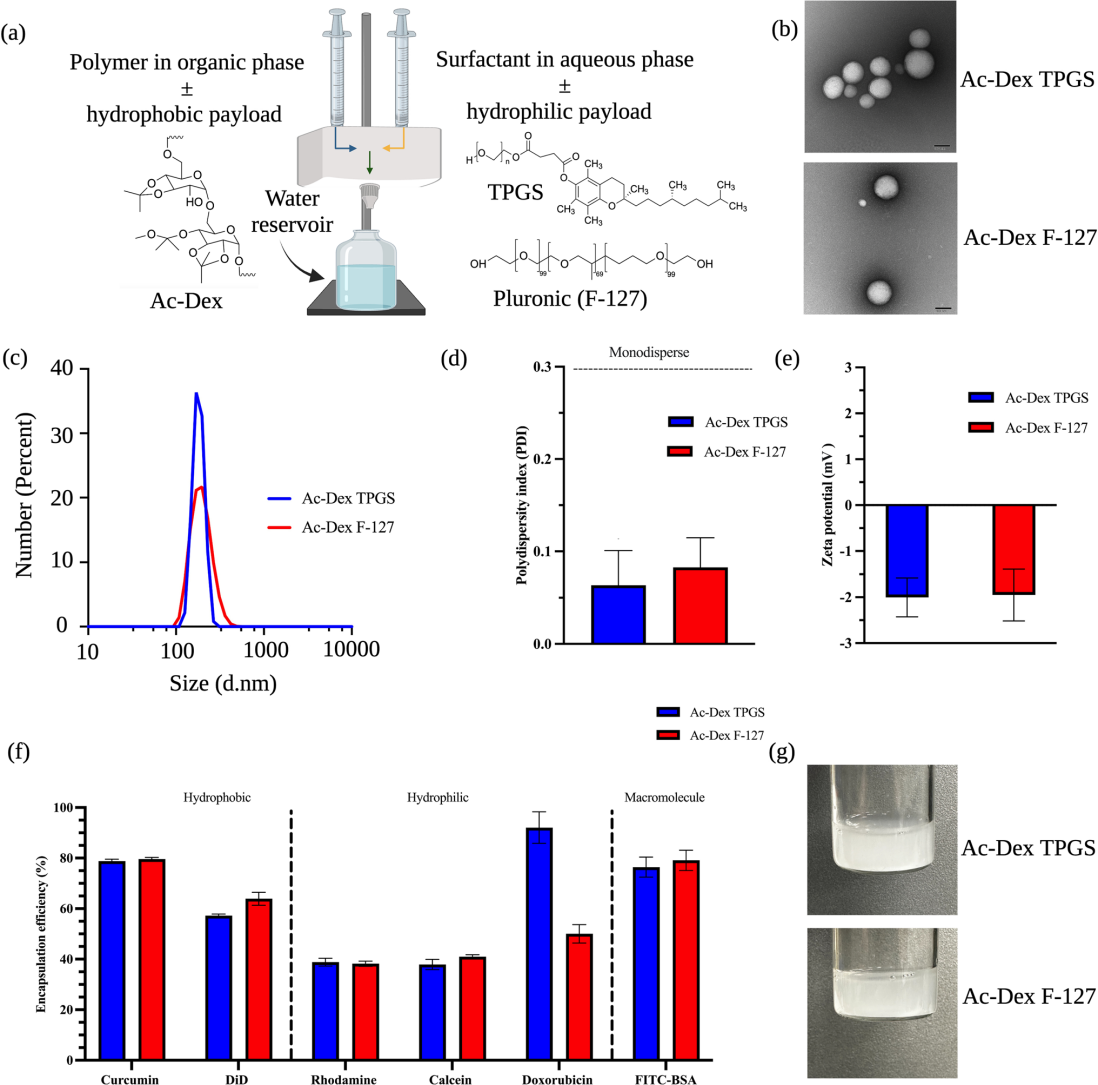
Formulation and subsequent physiochemical characterization of Ac-Dex polymeric nanoparticles stabilized by nonionic surfactants TPGS and F-127. **a** Schematic of the confined impinging jet mixer (CIJ), which forms polymeric nanoparticles through flash nanoprecipitation (FNP). The organic and aqueous phases and other compounds are represented with chemical structures included. The water quencher reservoir represents the quencher bath. **b** Transmission electron microscopy (TEM) images of Ac-Dex TPGS and Ac-Dex F-127 nanoparticles (Scale bar = 100 nm). **c** The size characterization of Ac-Dex nanoparticles through dynamic light scattering (DLS) was reported as a number percentage. **d** Polydispersity index (PDI) was obtained through DLS. No statistical difference exists between either Ac-Dex formulation as determined by an unpaired t-test. **e** Zeta potential was determined for the surface charge of the nanoparticle through electrophoretic light scattering (ELS). No statistical difference exists between Ac-Dex formulation as determined by an unpaired t-test. **f** Encapsulation efficiency (EE%) was determined for hydrophobic, hydrophilic, and protein-based macromolecules. **g** Images are provided showing the nanoparticles immediately after formulation. Data presented as mean ± s.d. (n = 3). The image was created in BioRender

FNP has been shown to rapidly fabricate nanoparticles with high reproducibility [23]. Ac-Dex nanoparticles that are often manufactured using an emulsification process utilize polyvinyl alcohol as the stabilizer [33]. PEGylated non-ionic surfactants are widely utilized to stabilize hydrophobic drugs or nanoparticles using the FNP technique. In addition, PEGylated nanoparticles have been shown to exhibit enhanced systemic circulation times as compared to non-PEGylated nanoparticles [34]. We hypothesized that hydrophobic Ac-Dex nanoparticles could be rapidly stabilized by the FNP technique using PEGylated surfactants. To validate this approach, we have chosen two non-ionic PEGylated surfactants, TPGS and F-127 as stabilizers. TPGS or F-127 stabilized Ac-Dex nanoparticles were formulated in a CIJ mixer, as seen in Fig. 1a. The polymer, such as ethanol, was dissolved in the organic phase, while the surfactants (TPGS or F-127) were introduced in the aqueous phase [35]. These two phases in a 1:1 v/v ratio simultaneously impinged in the CIJ mixer to undergo rapid turbulent mixing. This rapid mixing in the CIJ mixer allows supersaturated conditions and subsequent precipitation of hydrophobic components [22]. The resultant mixture was collected in a reservoir to quench the precipitation reaction, thus reducing the aggregation of nanoparticles.

Ac-Dex nanoparticles formulated using TPGS or F-127 were found to be morphologically stable with a spherical shape, as verified using transmission electron microscopy (TEM) (Fig. 1b). Additional TEM images can be found in the supplementary information in Fig. S1. Dynamic light scattering analysis confirmed that the sizes of Ac-Dex TPGS and Ac-Dex F-127 nanoparticles were 182 ± 27 d.nm and 199 ± 55 d.nm, respectively (Fig. 1c). The polydispersity index was found to be less than 0.15, indicating a highly monodisperse nature of these nanoparticles (Fig. 1d). Ac-Dex TPGS and Ac-Dex F-127 nanoparticles possess a neutral surface charge (Fig. 1e), which represents the successful stabilization of nanoparticles with non-ionic surfactants. A 1% w/v surfactant concentration produced monodispersed particles with high reproducibility (Table S1), and therefore, this concentration was chosen for all our experiments. Furthermore, Ac-Dex TPGS and F-127 nanoparticles formulated using ethanol through FNP were comparable to those formulated using a traditional double emulsion method utilizing chloroform, as seen in Figs. S2, S3, and S4. This signifies the importance of the rapid fabrication approach by FNP to generate Ac-Dex nanoparticles utilizing non-toxic solvents.

Various water-miscible organic solvents have been utilized to form diverse nanoparticles through the FNP process [36–41]. We were interested in understanding the suitability of different organic solvents besides ethanol to form Ac-Dex nanoparticles using the FNP technique. To perform these experiments, we have selected methanol, THF, acetone, DMF, and DMSO as solvents (Table 1). Interestingly, we saw an increase in the size of Ac-Dex TPGS or Ac-Dex TPGS particles with these solvents as compared to ethanol. Specifically, methanol, DMF, and DMSO formed particles larger than 500 nm, while acetone and THF formed particles smaller than 500 nm. Additionally, we explored the impact of reduced polymer concentration with various solvents, as shown in Table S2. We found that changing the polymer concentration does change the particle characteristics with different solvents that are used; it is likely that each solvent would need to have an individually optimized polymer concentration. This is an interesting finding because the size of Ac-Dex particles could be tuned with the choice of organic solvents, and modulating particle size may significantly impact their usage for different biomedical applications; we believe this phenomenon should be explored further in future works.

**Table 1:** Organic solvent utilized during FNP modulates the size of Ac-Dex TPGS and Ac-Dex F-127 nanoparticles

	Ac-dextran TPGS			Ac-dextran F-127		
	Size (d.nm)	PDI	Zeta potential (mV)	Size (d.nm)	PDI	Zeta potential (mV)
Ethanol	164.4 ± 76.1	0.2	− 1.93	196.8 ± 89.1	0.21	− 0.51
Methanol	500.1 ± 86.9	0.03	− 1.83	734.4 ± 183.5	0.06	− 0.06
Acetone	287.8 ± 72.7	0.06	− 2.42	318.3 ± 102.8	0.1	− 0.97
THF	419.3 ± 89.9	0.05	− 2.97	400.6 ± 87.0	0.05	− 1.24
DMSO	982.4 ± 264.0	0.07	− 2.13	901.8 ± 148.9	0.03	2.31
DMF	892.1 ± 265.0	0.09	0.05	2189.7 ± 564.4	0.06	− 1.44

The removal of volatile solvents all remained evaporations while the non-volatile solvents were removed via dialysis (3000 kDa). Size was determined through number-average diameter (d.nm) and polydispersity index (PDI) determined by DLS, and zeta potential was determined through ELS. Size (d.nm) is reported as mean ± s.d. (n = 3)

Next, we have chosen small molecules with hydrophobic (curcumin and DiD dye) or hydrophilic (doxorubicin, calcein, and rhodamine) properties and a macromolecule to validate the encapsulation ability of Ac-Dex nanoparticles. Hydrophobic molecules curcumin (LogP: 3) and DiD (LogP: > 6) were able to encapsulate into Ac-Dex nanoparticles (Fig. 1f). On the other hand, the cationic and anionic hydrophilic molecules rhodamine and calcein demonstrated comparable encapsulation efficiencies (~ 40%) in Ac-Dex TPGS and Ac-Dex F-127 nanoparticles. In addition, a widely studied anti-cancer drug, doxorubicin hydrochloride, and a model protein, fluorescein isothiocyanate conjugated to bovine serum albumin (FITC-BSA), were efficiently encapsulated into Ac-Dex nanoparticles (Fig. 1f). Overall, Ac-Dex nanoparticles could encapsulate and retain payloads with diverse physicochemical properties (Fig. 1g).

### Lyophilized Ac-Dex TPGS nanoparticles are highly stable than Ac-Dex F-127 nanoparticles

Nanoparticles stored in aqueous solvents could lead to physical instability, posing issues for long-term storage [42]. To overcome this issue, we next studied the suitability of Ac-Dex nanoparticles for lyophilization and evaluated their size characteristics and payload retention after storage. Cryoprotectants are required to be added to nanoparticle formulations to retain structural integrity during lyophilization [42]. During lyophilization, polymeric nanoparticles stabilized with surfactants can have some intrinsic protective qualities [43]. Our results show that additional cryoprotection is required, as there was a significant increase in particle size one-day post-lyophilization without a cryoprotectant (Fig. S5). Choosing an appropriate cryoprotectant is essential and must be optimized for each formulation type. We selected three widely studied cryoprotectants for nanoparticle preparations, trehalose, mannitol, and sucrose, to optimize the lyophilization procedure. Among different concentrations of cryoprotectants tested, 10% w/v of mannitol retained the size characteristics of Ac-Dex TPGS and F-127 formulations compared to their pre-lyophilization counterparts (Table S3).

**Fig. 2:**
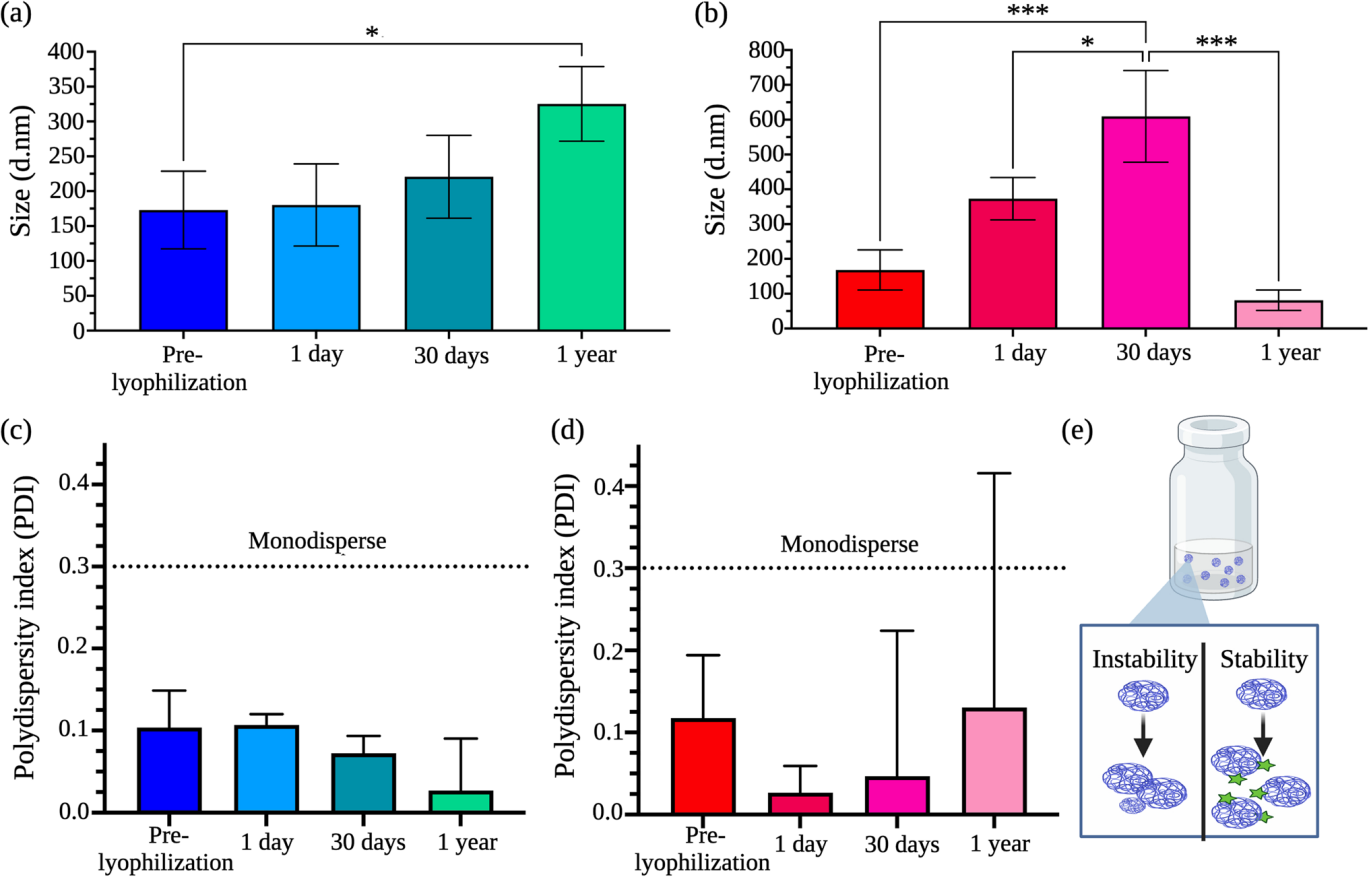
Ac-Dex TPGS formulations cryoprotected with 10% w/v mannitol demonstrated superior formulation stability after lyophilization and long-term storage at 4 °C. **a** Ac-Dex TPGS nanoparticles remained consistently sized for up to 1 year, and the size increase remained physiologically relevant for receptor-mediated endocytosis**.** The size characterization of Ac-Dex nanoparticles through dynamic light scattering (DLS) was reported as a number percentage. **b** Ac-Dex F-127 nanoparticles consistently increased in size for up to 1 year, when the size significantly decreased, suggesting particle instability-related collapse. The size characterization of Ac-Dex nanoparticles through DLS was reported as a number percent. **c** Ac-Dex TPGS nanoparticle formulations remained monodisperse for one year. The polydispersity index (PDI) was obtained through DLS. **d** Ac-Dex F-127 nanoparticle formulations remained monodisperse for up to 1 year when the PDI range expended, suggesting particle aggregation. PDI was obtained through DLS. **e** Schematic depicts the physical stability versus instability that can occur in nanoparticle formulations. The cryoprotectant may appropriately keep nanoparticles stable in size and monodisperse, or nanoparticles may remain unstable and collapse and aggregate**.** Statistical differences were determined by one-way ANOVA with Tukey's multiple comparison test. Data presented as mean ± s.d. (n = 3). The image was created in BioRender.com

Next, we evaluated the storage stability of lyophilized samples obtained using 10% w/v of mannitol as the cryoprotectant. For these studies, lyophilized cakes of Ac-Dex TPGS or Ac-Dex F-127 were stored in the refrigerator at 4 °C and hydrated with PBS at predetermined time intervals before analyzing their size. Interestingly, the Ac-Dex TPGS formulation showed no significant change in size on days 1 and 30 as compared to pre-lyophilization samples (Fig. 2a). A year after storage, the size of the particles increased from 173 ± 58.9 to 325 ± 54 d nm (Fig. 2a); however, the particles showed greater monodispersity at all time points with a PDI of < 0.2 (Fig. 2c). Of note, no significant difference in size was observed between the 1-day post-lyophilization and 1-year lyophilization samples. Additionally, the surface charge Ac-Dex TPGS nanoparticles remained within the neutral range (− 2.78 mV) after the lyophilization, which further supports that nanoparticles remain stable with long-term storage. On the other hand, the Ac-Dex F-127 formulation showed a significant increase in size with each time point tested. The size of nanoparticles increased from 168 ± 55.7 to 610 ± 132 d.nm after 30 days of storage (Fig. 2b). To our surprise, after 1-year storage, particles collapsed and aggregated with a significant drop in particle size (Fig. 2b). While the values were not statistically significant, there was an increase in PDI at one year that exhibited a high standard deviation (Fig. 2d), which might indicate a change in nanostructure integrity (Fig. 2e). We further verified whether Ac-Dex TPGS nanoparticles could retain hydrophobic (DiD) or hydrophilic (doxorubicin hydrochloride) payloads after lyophilization. Excitingly, DiD and doxorubicin hydrochloride showed 81% and 99% retention, respectively, following the rehydration of lyophilized cakes (Fig. S6). These results demonstrate that lyophilized Ac-Dex TPGS nanoparticles are highly stable and more capable of retaining encapsulated payloads. Given the high stability of Ac-Dex TPGS nanoparticles, we extensively studied them for all other in vitro cellular and in vivo experiments. For additional insight into the particle stability, we examined how the particle size and encapsulated payload would be impacted when Ac-Dex TPGS nanoparticles were exposed to a protein-rich environment (Fig. S7). When nanoparticles were incubated with 10% FBS for 72 h, we did not observe a significant change in the particle size as compared to the control (nanoparticles in only PBS). Furthermore, 77% of the DiD payload was slowly released over 72 h in FBS indicating no dramatic change in the morphological integrity of nanoparticles.

### Ac-Dex TPGS nanoparticles are inherently nontoxic and demonstrate time-dependent clathrin-mediated endocytosis

**Fig. 3:**
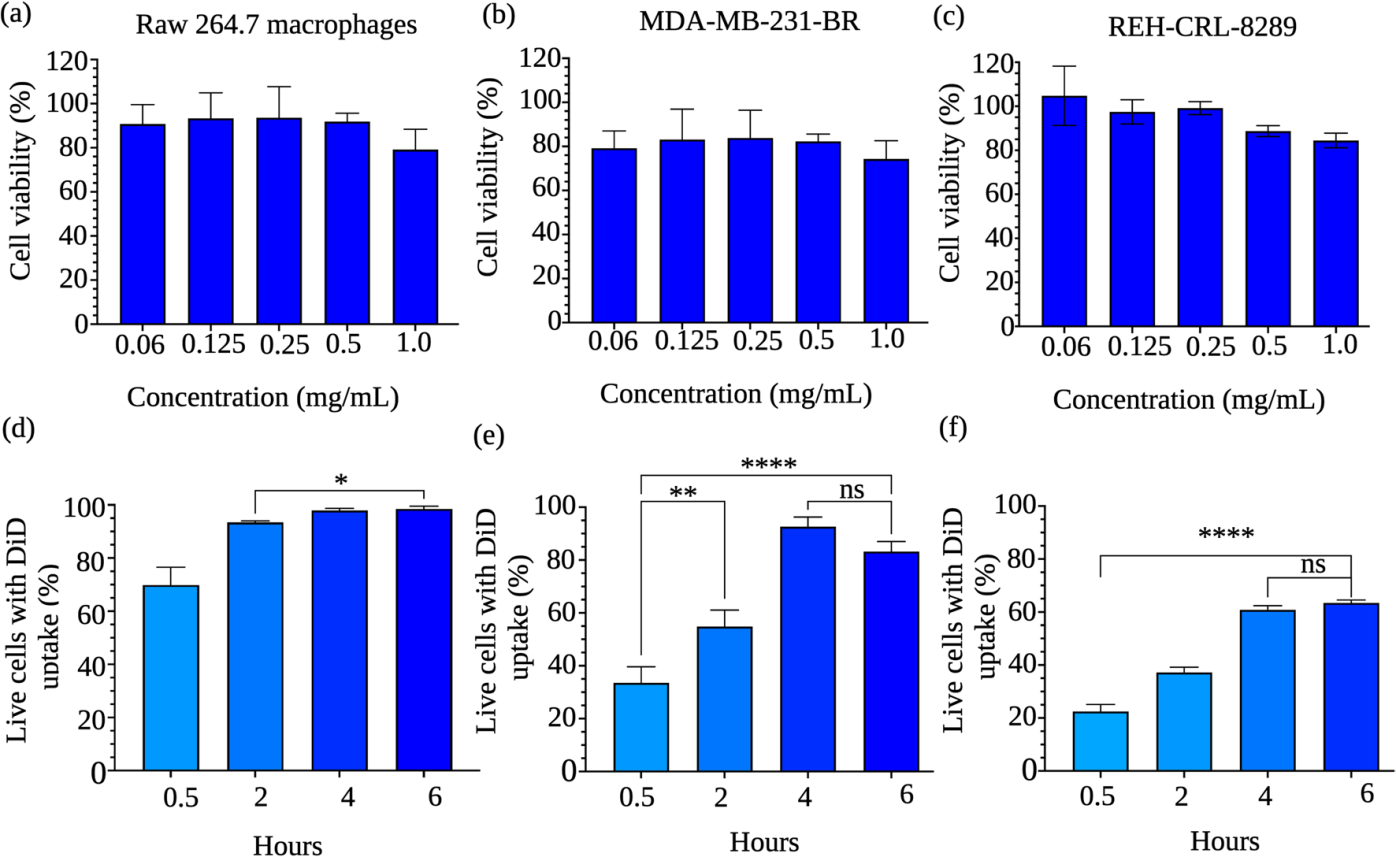
Acetalated-dextran TPGS formulations are inherently nontoxic in multiple cell lines and undergo time-dependent endocytosis. Concentrations up to 1 mg/mL were used to determine toxicity through a 48-h MTT assay; all concentrations tested exhibited viability > 70%. PBS controls were used to determine baseline cell viability compared to nanoparticle-treated wells **a** Raw 264.7 macrophages, **b** MDA-MD-231-BR cells, **c** REH-CRL-8286, **d** Time-dependent cellular uptake by Raw 264.7 macrophages. **e** Time-dependent cellular uptake by MDA-MD-231-BR cells. **f** Time-dependent cellular uptake by REH-CRL-8286 cells. Statistical differences were determined by one-way ANOVA with Tukey's multiple comparison test (****p < 0.0001) Data was reported as a mean ± s.d. (n = 4)

Ac-Dex nanoparticles formulated using emulsion-based methods and PVA as the stabilizer have previously been shown to be biocompatible, allowing efficient cellular delivery of payloads [12, 19, 44]. We have chosen a primary immune cell line, Raw 264.7 macrophages, to establish the inherent toxicity of blank Ac-Dex TPGS nanoparticles and cellular uptake kinetics. The nanoparticles were incubated with cells at various concentrations of up to 1 mg/mL of Ac-Dex for 48 h, and cell viability was measured using the MTT assay. The viability threshold for toxicity according to ISO 10,993–5 is often < 70% for a compound to be considered cytotoxic [45, 46]. At all tested concentrations, the Ac-Dex TPGS nanoparticles were non-toxic and demonstrated cell viability greater than 75% (Fig. 3a). Of note, we also observed that Ac-Dex F-127 nanoparticles were nontoxic, with viability > 75% (Fig. S8). A similar non-toxic behavior was observed in adherent solid tumor cells (MDA-MB-231-BR triple-negative breast cancer), and nonadherent liquid tumor cells (REH-CLR-8286 B-cell acute lymphocytic leukemia), as seen in Fig. 3b, c.

Ac-Dex TPGS nanoparticles were encapsulated with a fluorescent DiD dye for cellular uptake studies, and the percentage of DiD-positive (Fig. 3d–f) cells at each time point was measured using flow cytometry. A significant increase in cellular uptake between each time point was observed until 2 h. However, a plateau was attained after 2 h with no further significant change in the uptake. Time-dependent cellular uptake was also observed with MDA-MD-231-BR and REH-CRL-8286 cell lines, with an 83 and 64% uptake, respectively, after 6 h. For Raw 264.7 macrophages, no significant change in the cellular uptake was observed at 24 h as compared to 6 h whereas REH-CRL-8289 cells demonstrated a significant change in the cellular uptake at 24 h as compared to 6 h (Fig. S9). These results indicate differential cellular uptake abilities of Ac-Dex TPGS nanoparticles based on the type of cell line. Ac-Dex F-127 nanoparticles also showed a significant change in cellular uptake with time when tested in macrophages, although a lower cellular uptake was evident overall as compared to Ac-Dex TPGS nanoparticles (Fig. S10).

To understand the mechanism of cellular uptake of Ac-Dex nanoparticles, we treated macrophages with a clathrin-dependent endocytic inhibitor, chlorpromazine (CPZ), and caveolae-mediated endocytic inhibitor, genistein before incubating them with nanoparticles (Fig. S11). Interestingly, there was a significant reduction in cellular uptake with CPZ inhibition, with only 49 ± 2.4% of cells having endocytosed the DiD-loaded Ac-Dex TPGS nanoparticles, compared to the uninhibited 6-h uptake in which 92% ± 2% of cells are DiD positive. There was no significant reduction in cellular uptake observed with genistein inhibition as compared to the uninhibited 6-h uptake. This result confirms that the Ac-Dex nanoparticles utilize a clathrin-dependent pathway of endocytosis.

### Ac-Dex nanoparticles selectively release payloads in acidic environments with high-precision

**Fig. 4:**
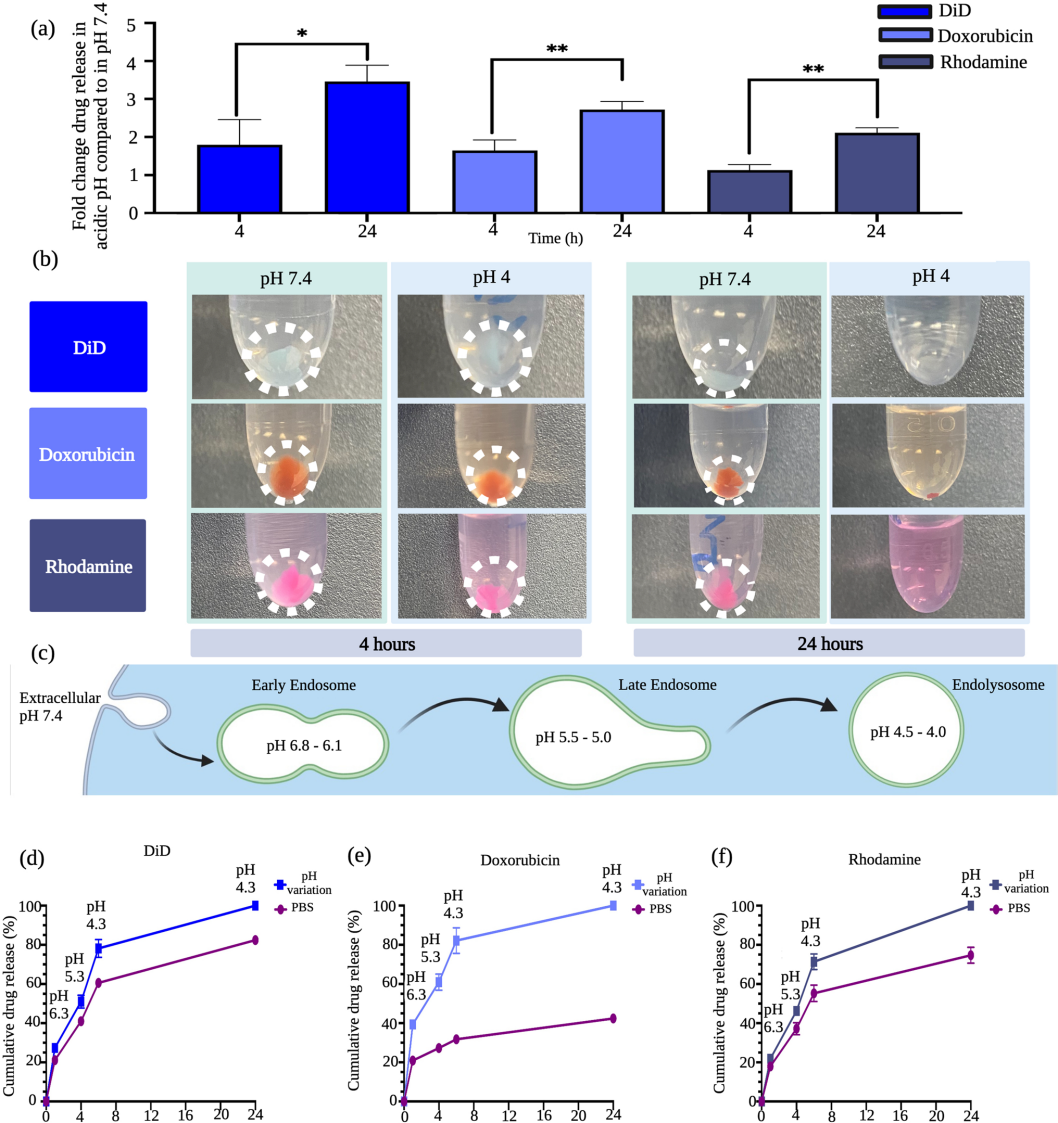
Ac-Dex TPGS formulations are pH-responsive, releasing more of the encapsulated payload at an acidic pH than at a physiological pH. **a** Ac-Dex TPGS nanoparticles release more hydrophilic and hydrophobic payloads at 4 and 24 h at a pH of 4 vs. payload release in a control PBS solution of pH 7.4. Statistical differences were determined by nonpaired t-tests (*p < 0.05, **p < 0.005). **b** Visual images demonstrate the degradation of the polymer pellet over time as the nanoparticle is dissolved in an acidic pH media. At 4 h, the pellets are still visible in both the pH 4 and 7.4 solutions; however, as time progresses, the supernatant becomes more saturated with the chromophore, and the pellet decreases in the acidic medium. **c** Schematic representative of the endolysosomal pH changes that occur when a nanoparticle is internalized. After endocytosis and entrapment in the early endosome, the internalization process transitions to the increasingly acidic late endosome. The late endosome then undergoes fusion with lysosomes, which again causes more acidification of the environment. **d**–**f** Ac-Dex TPGS nanoparticles, when exposed to pH changes that mimic the endolysosomal pathway, release more of the hydrophilic and hydrophobic payloads compared to the release that occurs in a control solution of PBS with a pH of 7.4. Data was reported as a mean ± s.d. (n = 3). Image was created using BioRender.com

Ac-Dex nanoparticles were reported to be stable at physiological pH by only releasing the majority of their encapsulated payloads in acidic environments such as cellular endolysosomes [2, 16]. Our Ac-Dex TPGS nanoparticles demonstrated a greater release of payloads in acidic conditions (pH 4) than at a physiological pH 7.4, demonstrating the pH-responsive nature of nanoparticles (Fig. 4a, b). We demonstrated the release of both hydrophilic (rhodamine and doxorubicin) and hydrophobic (DiD) molecules, which all had statistically higher release of payloads after 24 h in the acidic environment. After 4 h of incubation, Ac-Dex nanoparticles showed a 1.8–3.5-fold change in the release of DiD, a 1.6–2.7-fold change in the release of doxorubicin, and 1.1-to-2.1-fold change in the release of rhodamine in acidic pH of 4 as compared to the physiological pH 7.4 (Fig. 4a) and this was also evident with a change in nanoparticle pellet size following centrifugation (Fig. 4b). Interestingly, the dissolution of nanoparticles was achieved in 24 h at an acidic pH, allowing 100% release of payloads (Fig. 4a, b). This dissolution could be attributed to the complete conversion of hydrophobic acetal dextran to hydrophilic dextran by breakage of acetal bonds at low pH [47]. Endolysosomal pH release represented as fold change for Ac-Dex F-127 nanoparticles also followed similar trends as Ac-Dex TPGS nanoparticles (Fig. S12).

As nanoparticles are endocytosed, they are exposed to an increasingly acidic pH through the endolysosomal pathway, as represented in Fig. 4c. The time from early endosome to late endosome can vary; however, it has been shown that cargo can often stay within an early endosome for up to 1 h before transitioning to a late endosome [48]. We explored the relationship between the cumulative release of a payload and the gradual acidification over time. We found that after exposure to pH 6.3 for 1 h, the Ac-Dex TPGS nanocarriers released 27% DiD of the total payload compared to only releasing 21% when exposed to PBS with physiological pH for the same time (Fig. 4d). For doxorubicin, a similar trend was observed with 39% when in pH 6.3 compared to 20% release in PBS (Fig. 4e). Rhodamine was not an exception to this trend, showing 21% release in pH 6.3 versus 17% in PBS (Fig. 4f).

After the transition to the late endosome, the cargo can be exposed to a pH between 5.5 and 5.0 for several hours before transitioning into the lysosome [49]. Our results show that for 4 h, the nanoparticles at pH 5.3 released 50% DiD, 60% doxorubicin, and 46% rhodamine of the total payload, while the control group with PBS released 40%, 27%, and 37%, respectively. After the late endosome, the cargo transitions to the endolysosome, introducing an even more acidic environment. We show that at 6 h, nanoparticles at pH 4.3 released 78% DiD, 82% doxorubicin, and 71% rhodamine of the total payload, while in comparison, the PBS group only released 60%, 31%, and 55%, respectively.

We continue this study until 24 h, expecting nanoparticle residence in endolysosomal conditions. In agreement with our other release study, the nanoparticles at pH 4.3 showed a total release after 24 h as compared to physiological pH, which had 82% release in DiD, 42% payload release in doxorubicin, and 75% payload release in rhodamine. It is important to note that to simulate the pH incubation conditions exactly, we exposed the same nanoparticles for each pH treatment through the entire pH range (6.3–4.3). Of note, it is important to state that diffusion will happen through hydrolysis even at physiological pH, the advantage of Ac-Dex nanoparticles is that this hydrolysis happens at an accelerated rate which also has the advantage of tunability.

### Ac-Dex nanoparticles permit cytosolic release in multiple cell lines.

**Fig. 5:**
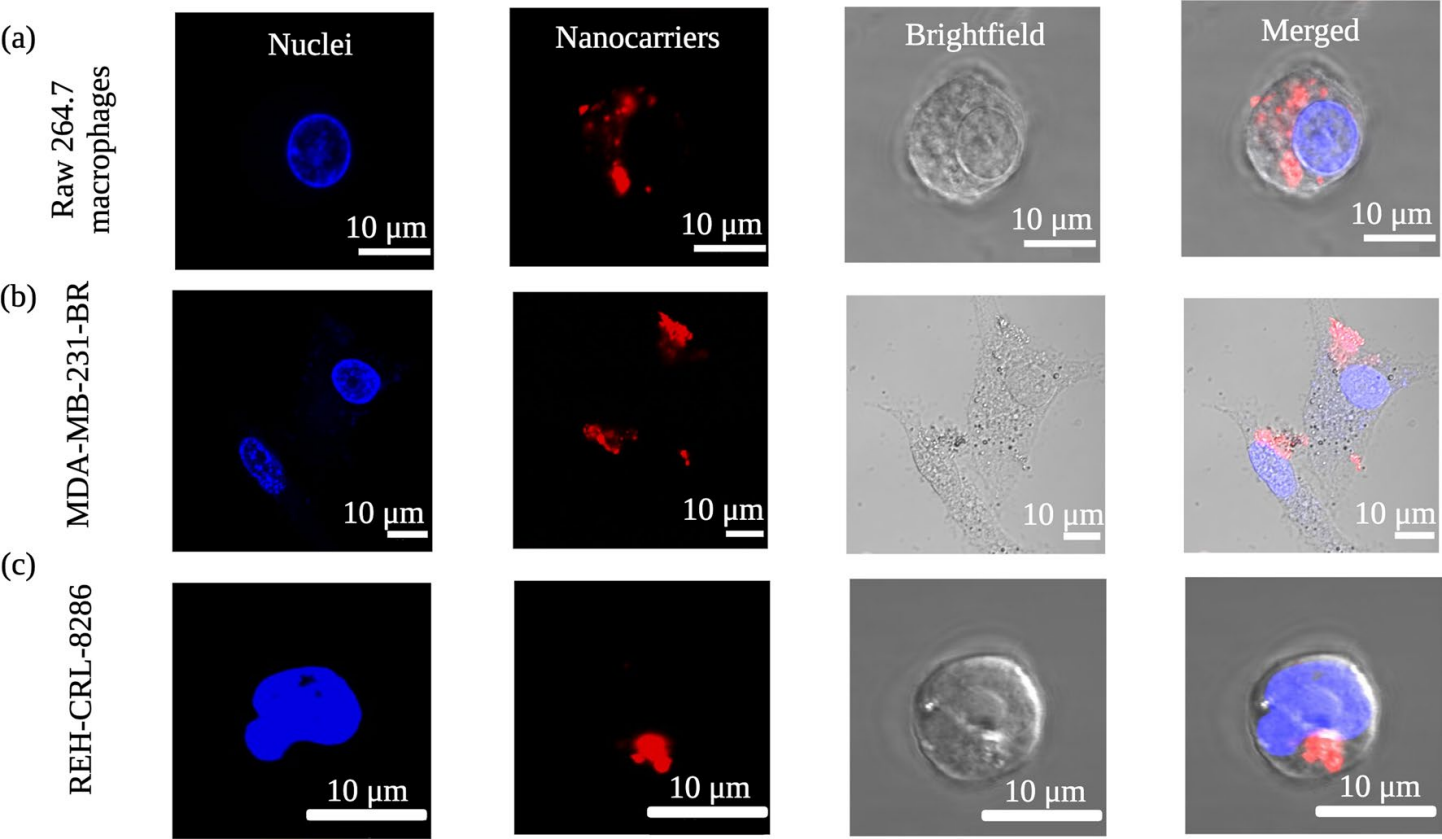
Intracellular delivery and subsequent cytosolic release by Ac-Dex TPGS nanoparticles loaded with DiD at 24 h. Each panel differentiates the nuclei (blue color), the DiD nanoparticles (red color), the brightfield showing the cell membrane, and the merged image of each channel overlay. Live cell images were taken with the 60X objective, and cytosolic release is indicated inside the cell as diffused red fluorescence. The scale bar is 10 µm. **a** Raw 264.7 macrophages, **b** MDA-MB-231-BR cells, and **c** REH-CRL-8286 cells

After cellular internalization, the nanoparticles still must release their payloads within the cytosol to show the desired therapeutic effects. To demonstrate this, we incubated DiD-loaded nanoparticles with three different cell lines: Raw 264.7 macrophages, MDA-MD-231-BR, and REH-CRL-8286 for 24 h and monitored the cytosolic release through confocal microscopy. Interestingly, a diffused, rather than punctate, which is often representative of endolysosomal trapping, red fluorescence from the encapsulated DiD dye was observed in the cytoplasm of all three cell lines (Fig. 5a–c). These results demonstrate that Ac-Dex TPGS nanoparticles stimulated under acidic endolysosomal conditions could release payloads into the cytoplasm efficiently. Additionally, we have evaluated endolysosomal release using a hydrophilic dye as well, which conveys similar results (Fig. S13). Of note, Ac-Dex F127 nanoparticles could also release payloads in the cytoplasm when tested in the REH-CRL-8286 cell line (Fig. S14).

### Intravenously administered Ac-Dex TPGS nanoparticles are well tolerated with greater biodistribution in the liver and spleen at 4 h and lower organ accumulation at 24 h

The mononuclear phagocyte system (MPS) is responsible for the clearance of nanoparticle-based therapeutics that are delivered intravenously. This multiorgan system involves the liver, spleen, lungs, and kidneys [50, 51]. The MPS system contributes to the clearance of nanoparticles from systemic circulation, thus limiting nanoparticle development for drug delivery applications [52]. Most commonly, nanoparticles are seen to accumulate in MPS organs, specifically in the spleen and liver [53]. The goal does not always need to be avoiding the MPS entirely; the spleen is particularly interesting to target with cancer immunotherapies [50, 54]. Multiple cell types, including B cells, T cells, macrophages, monocytes, and dendritic cells, can all be found within the spleen, facilitating the modulation of immunotherapies [50], and this makes the spleen especially enticing for drug delivery. The liver can also be advantageous to target with nanoparticles due to the immune response associated with Kupffer cells, which may be useful for therapeutics for autoimmune disorders [55].

**Fig. 6:**
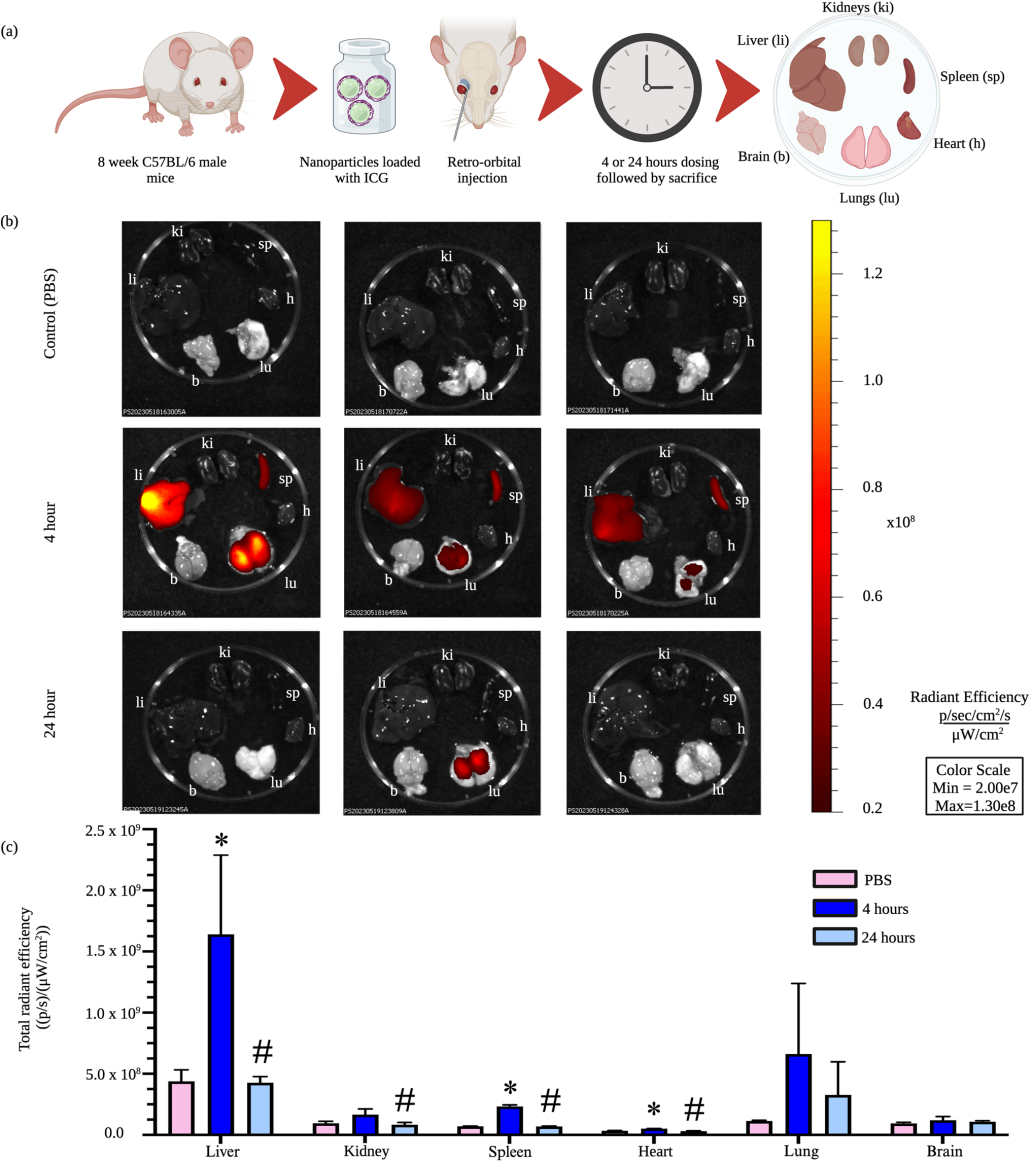
In vivo delivery of Ac-Dex TPGS nanoparticles in C57BL/6 mice. **a** Schematic depicting ICG-loaded Ac-Dex TPGS nanoparticles intravenously administered followed by IVIS imaging of the organs. **b** IVIS imaging of the kidneys (ki), spleen (sp), heart (h), lungs (lu), brain (b), and liver (li) of controls, 4 h nanoparticle exposure (n = 3) and 24 h nanoparticle exposure from Ac-Dex TPGS nanoparticles (n = 3). PBS exposure is also included for control comparison (n = 3). Each panel is a replicate of the treatment duration stated on the left. Fluorescence is presented as total radiant efficiency. **c** The liver, kidney, spleen, and heart showed significant differences compared to the control group. Significance was determined by ordinary one-way ANOVA with Tukey’s multiple comparison. Data are presented as mean ± s.d. (n = 3) (*p < 0.05 vs. PBS, and #*p* < 0.05 vs. 4 h)

Ac-Dex has previously been shown to effectively deliver immunological adjuvants and small-molecule therapeutics, and numerous immunomodulatory applications should be further studied, including autoimmune diseases and cancer vaccines [19]. To determine the organ-level biodistribution of Ac-Dex TPGS nanoparticles following intravenous administration, we encapsulated indocyanine cyanine green (ICG) and measured fluorescence through IVIS imaging. The ICG-loaded nanoparticles were intravenously administered through the retro-orbital plexus of C57BL/6 J male mice. The various organs (liver, spleen, kidney, lungs, heart, and brain) were harvested either at 4 or 24 h (Fig. 6a). The total radiant efficiency was quantified for each organ (Fig. 6b). Ac-Dex TPGS nanoparticles primarily had uptake in the liver at 4 h, as well as an increased uptake in the spleen and lungs compared to other organs (Fig. 6c). The 4 h biodistribution into MPS could be attributed to faster cellular uptake of Ac-Dex TPGS nanoparticles by phagocytic cells, which agrees with in vitro uptake studies.

Interestingly, while not significant due to high variation, uptake was observed in the lungs at 4 h, although the fluorescent signal is largely absent after 24 h. The initial fluorescence signal could be indicative of nanoparticle accumulation within the lung. This accumulation within the lungs could be concerning as long-term accumulation has been shown to lead to lung complications such as asthma or chronic obstructive pulmonary diseases [56]. These nanoparticles are unlikely to lead to alveolar damage due to the vast signal reduction that has been seen at 24 h time point. Ac-Dex has previously been useful for pulmonary delivery, which utilizes the acidic pH microenvironment of the pulmonary mucosa to support controlled degradation [57]. The pH-responsiveness of the Ac-Dex nanoparticle is what allows for this controlled degradation; this factor makes it a promising drug delivery system, as polymer accumulation can be avoided through tunable degradation. Incorporating targeting motifs could be additionally promising as a drug delivery system and should be explored further. At 24 h, there were significant reductions in uptake in the liver, kidney, spleen, and heart compared to 4 h, indicating clearance and degradation of Ac-Dex TPGS nanoparticles. This is an interesting phenomenon, as the degradation of nanoparticles at later time points would allow frequent administrations of nanotherapeutics with minimal burden of polymeric excipient accumulation in different organs.

## Conclusion

Here, we developed an FNP approach to fabricate Ac-Dex nanoparticles easily using a non-toxic solvent such as ethanol. The CIJ mixer allowed rapid stabilization of hydrophobic Ac-Dex nanoparticles using PEGylated non-ionic surfactants, TPGS, and F-127. The size of Ac-Dex particles could be modulated using FNP from 200 nm to 2 µm by varying the organic solvent and surfactant type. From a biomedical application standpoint, size modulation is a critical aspect. Ac-Dex nanoparticles formed using TPGS and F-127 were able to encapsulate a wide range of payloads with diverse physicochemical properties. The Ac-Dex TPGS nanoparticles formed through FNP were highly stable for up to a year when lyophilized using mannitol as a cryoprotectant. The lyophilized nanoparticles were able to retain hydrophobic and hydrophilic payloads with > 85% efficiency.

Ac-Dex nanoparticles were inherently non-toxic to cells and showed time-dependent cellular uptake in different cell lines. Ac-Dex nanoparticles were highly responsive to acidic pH, and in simulated endolysosomal conditions, encapsulated payloads released faster as compared to physiological pH. This showcases the ability of these nanoparticles to have minimal leakage of encapsulated payloads in extracellular conditions. Furthermore, Ac-Dex TPGS nanoparticles precisely released all the encapsulated payloads into the cytoplasm of the cells. Intravenously administered Ac-Dex TPGS nanoparticles were well tolerated and majorly biodistributed to the liver, spleen, and lungs. These nanoparticles cleared within 24 h, indicating a lower chance of organ-level accumulation. It is important to note that we exploited the in vitro and in vivo abilities of Ac-Dex TPGS nanoparticles using model fluorophores. Future studies warrant in-depth optimization for different drug payloads to achieve the desired therapeutic effects to the fullest.

Overall, we demonstrated a simple, rapid, and scalable approach to forming Ac-Dex nanoparticles. This non-exothermic way of fabricating nanoparticles highly suits the encapsulation of thermolabile drugs, protein therapeutics, and vaccines. Of note, employing a non-toxic solvent such as ethanol, which is often used in the fabrication of clinically dispensed lipid nanoparticle formulations, would help further the development of Ac-Dex nanoparticles.

### Supplementary Information

**Additional file 1**. Nanoparticle characterization and in vitro cell analysis.

## Data Availability

The datasets generated are available from the corresponding author upon reasonable request.
