# Supplementary material for: Flash nanoprecipitation allows easy fabrication of pH-responsive acetalated dextran nanoparticles for intracellular release of payloads
Source: Discov Nano. 2024 Jan 4;19(1):4. doi: 10.1186/s11671-023-03947-w (PMC10766584; doi:10.1186/s11671-023-03947-w)
Supplement: Supplementary file 1 — Additional file 1. Nanoparticle characterization and in vitro cell analysis. [file 11671_2023_3947_MOESM1_ESM.docx]

**Supplementary Material**

**Flash nanoprecipitation allows easy fabrication of pH-responsive acetalated dextran nanoparticles for intracellular release of payloads.**

Krystal A. Hughes^1^, Bishal Misra^1^, Maryam Maghareh^2^, Parinya Samart^1,3^, Ethan Nguyen^1^, Salik Hussain^4,5^, Werner J. Geldenhuys^1,6^, Sharan Bobbala^1^

1 Department of Pharmaceutical Sciences, West Virginia University School of Pharmacy, Morgantown, WV, 26505

2 Department of Clinical Pharmacy, West Virginia University School of Pharmacy, Morgantown, WV, 26505

3 Siriraj Center of Excellence for Stem Cell Research, Faculty of Medicine Siriraj Hospital, Mahidol University, Bangkok, Thailand

4 Department of Microbiology, Immunology & Cell Biology, West Virginia University School of Medicine, Morgantown, WV, 26505

5 Department of Physiology, Pharmacology & Toxicology, West Virginia University, Morgantown, WV, 26505

6 Department of Neuroscience, West Virginia University School of Medicine, Morgantown, WV, 26505

Address correspondence to Sharan Bobbala, [sharan.bobbala@hsc.wvu.edu](mailto:sharan.bobbala@hsc.wvu.edu)

| **Table S1** Compositions of polymer and PEGylated nonionic surfactants utilized for the optimization process. Size (d.nm) and polydispersity index (PDI) determined by DLS, and zeta potential was determined through ELS. Size (d.nm) is reported as mean ± s.d. (n=3) | | | | | | |
| --- | --- | --- | --- | --- | --- | --- |
| Formulation | Ac-Dex (mg) | Surfactant W/V (%) | | Size (d.nm) | PDI | |
|  |  | TPGS | Pluronic |  |  | |
| 1 | 10 | 1 |  | 153 ± 35 | 0.05 | |
| 2 | 10 | 2 |  | 158 ± 37 | 0.06 | |
| 3 | 20 | 0.1 |  | 105 ± 26 | 0.06 | |
| 4 | 20 | 0.5 |  | 135 ± 59 | 0.19 | |
| 5 | 20 | 1 |  | 182 ± 27 | 0.02 | |
| 6 | 20 |  | 1 | 199 ± 55 | 0.08 | |
| 7 | 20 | 2 |  | 220 ± 52 | 0.05 | |
| 8 | 30 | 1 |  | 162 ± 58 | 0.126 | |
| 9 | 40 | 1 |  | 145 ± 75 | 0.27 | |

| **Table S2** Organic solvent utilized during FNP modulates the size of Ac-Dex TPGS nanoparticles at lower polymer concentrations. Methanol removal remained evaporation while DMSO was removed via dialysis (3000 kDa). Size was determined through number-average diameter (d.nm) and polydispersity index (PDI) determined by DLS. Size (d.nm) is reported as mean ± s.d. (n=3) | | |
| --- | --- | --- |
|  | Size (d.nm) | PDI |
| Methanol | 199 ± 85 | 0.18 |
| DMSO | 1026 ± 1340 | 1.7 |

| **Table S3** The process of cryoprotectant optimization compared to the initial physiochemical characteristics of Ac-Dex TPGS and F-127 formulations to the characteristics after lyophilization. Size (d.nm) and polydispersity index (PDI) determined by DLS. Size (d.nm) is reported as mean ± s.d. (n=3). | | | | | | | |
| --- | --- | --- | --- | --- | --- | --- | --- |
|  | Ac-Dextran TPGS | | | Ac-Dextran F-127 | | | |
| Cryoprotectant | W/V % | Size (d.nm) | PDI | W/V % | Size (d.nm) | PDI | |
| Initial | - | 196 ± 85.5 | 0.19 | - | 492 ± 117.2 | 0.05 | |
| Trehalose | 5 | 339 ± 125.9 | 0.13 | 5 | 423.6± 149.1 | 0.12 | |
| Sucrose | 5 | 316.3 ± 91.1 | 0.08 | 5 | 584.8 ± 109.9 | 0.03 | |
|  | 10 | 399.9 ± 92.8 | 0.05 | 10 | 292.9 ± 108.7 | 0.13 | |
|  | 15 | 344.9± 69.1 | 0.04 | 15 | 285.9 ± 79.7 | 0.07 | |
| Mannitol | 5 | 341.7 ± 55.1 | 0.02 | 5 | 372.9 ± 60.9 | 0.02 | |
|  | 10 | 165.4 ± 53.7 | 0.11 | 10 | 236.6 ± 94.8 | 0.16 | |
|  | 15 | 186.1± 59.4 | 0.11 | 15 | 276.9 ± 73.0484 | 0.06 | |
| **Figure S1** Transmission electron microscopy (TEM) images of Ac-Dex TPGS (a, and b) and Ac-Dex F-127 (c) nanoparticles (Scale bar = 200 nm) | | | | | | |  |
| 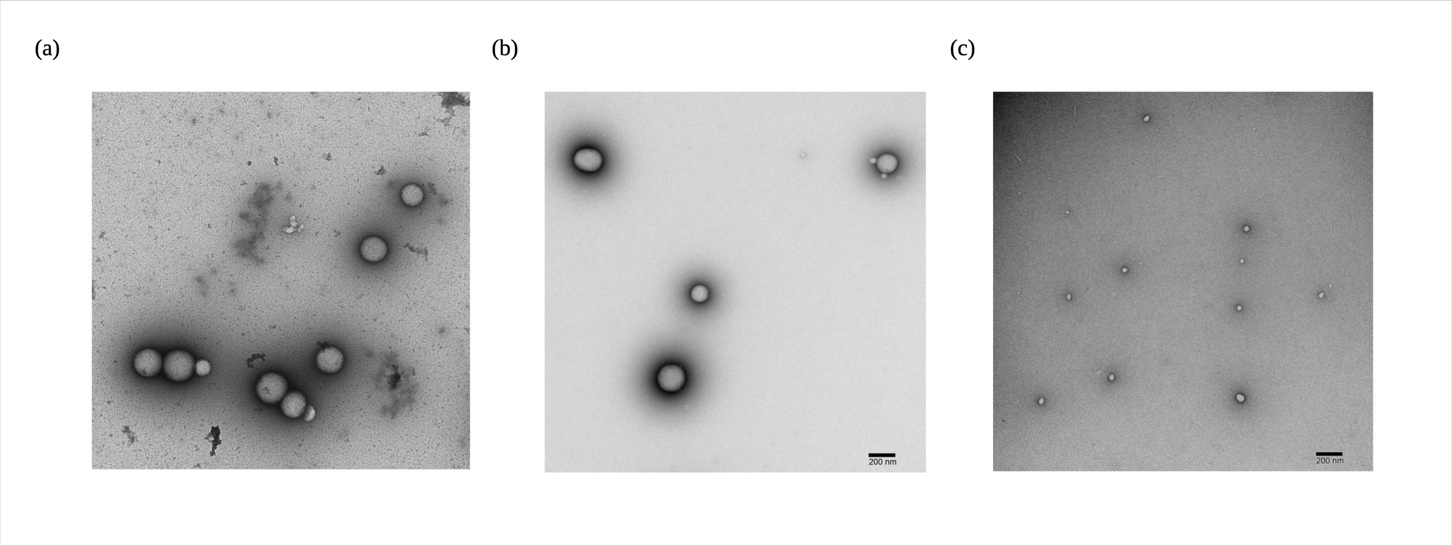 | | | | | | |  |

|  |
| --- |
| Address correspondence to Sharan Bobbala, sharan.bobbala@hsc.wvu.edu |

| **Figure S2** The size of Ac-Dex nanoparticles when formulated with TPGS or F-127 as surfactants are significantly smaller than traditionally prepared double emulsion-based formulation using PVA as a surfactant. Size (d.nm) is reported as mean ± s.d. (n=3). The statistics were evaluated using a one-way ANOVA with Tukey’s multiple comparison test. (** *p* < 0.01) |
| --- |
| 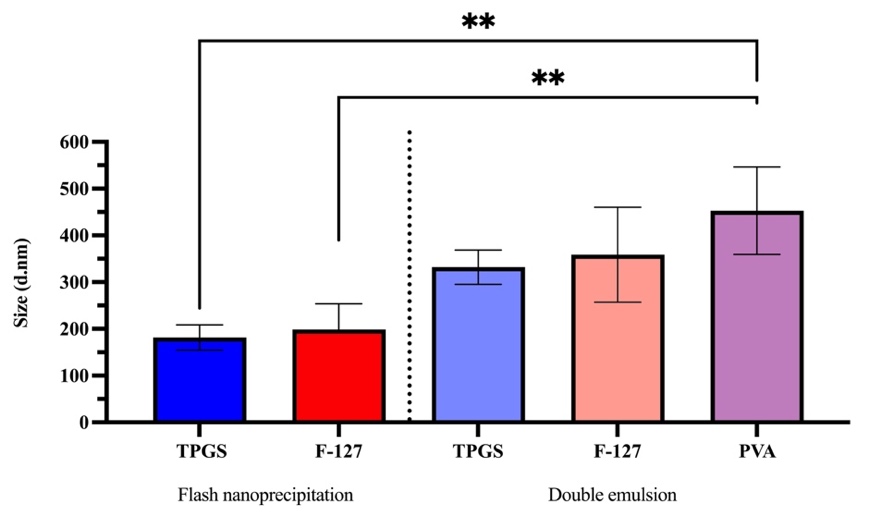 |

| **Figure S3** The polydispersity index (PDI) for all Ac-Dex formulations, regardless of surfactant choice had no significant differences between groups. The PDI characterization of Ac-Dex nanoparticles determined by dynamic light scattering (DLS). PDI is reported as mean ± s.d. (n=3). |
| --- |
| 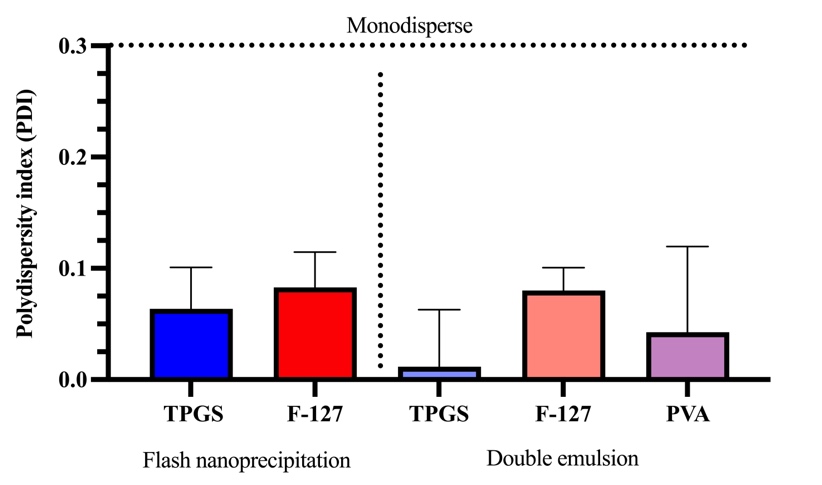 |

| **Figure S4** Encapsulation efficiency (EE%) for 1 mg of doxorubicin was determined for Ac-Dex formulations which were made either by flash nanoprecipitation (FNP) or double emulsion using a variety of surfactants. The Ac-Dex TPGS nanoparticles that were made via FNP had significantly more encapsulation of doxorubicin than all the formulations made through double emulsion. Data are reported as mean ± s.d. (n=3). The statistics were evaluated using a one-way ANOVA with Tukey’s multiple comparison test (**** *p* < 0.0001). |
| --- |
| 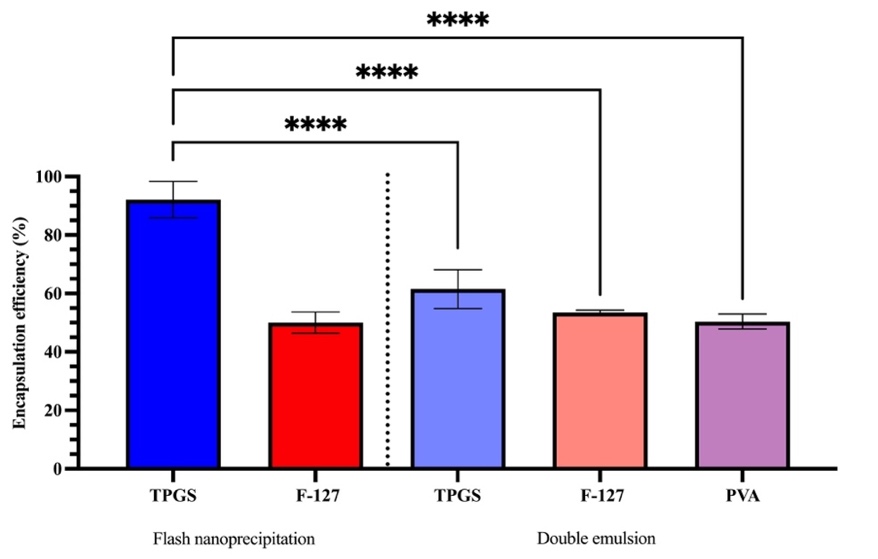 |

| **Figure S5** Role of cryoprotectant in the lyophilization of Ac-Dex nanoparticles. Mannitol (10% W/V) is used as the cryoprotectant. Size (d.nm) is reported as mean ± s.d. (n=3). The statistics was evaluated using a one-way ANOVA with Tukey’s multiple comparison test (** *p* < 0.001). |
| --- |
| 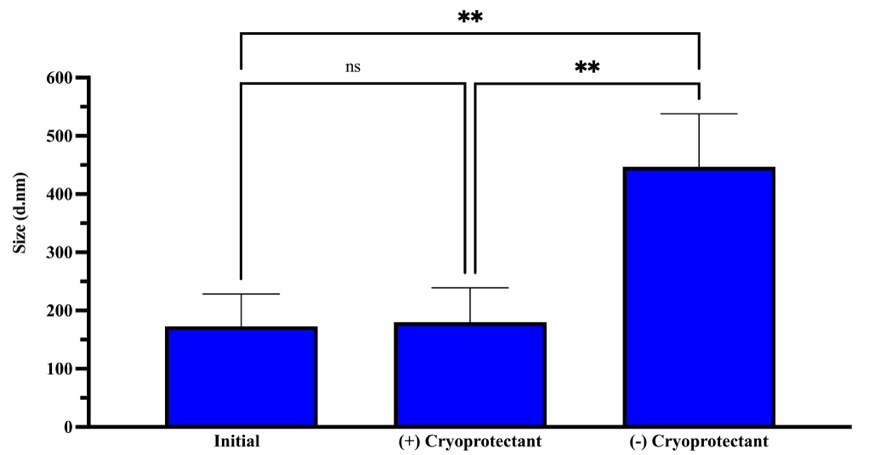 |

| **Figure S6** Ac-Dex TPGS lyophilized with 10% W/V mannitol retained hydrophilic and hydrophobic encapsulants when tested using DiD and Doxorubicin as model molecules. Lyophilized cakes were rehydrated one-day after the lyophilization with PBS. Retention of the encapsulant is reported as mean ± s.d. (n=3). |
| --- |
| 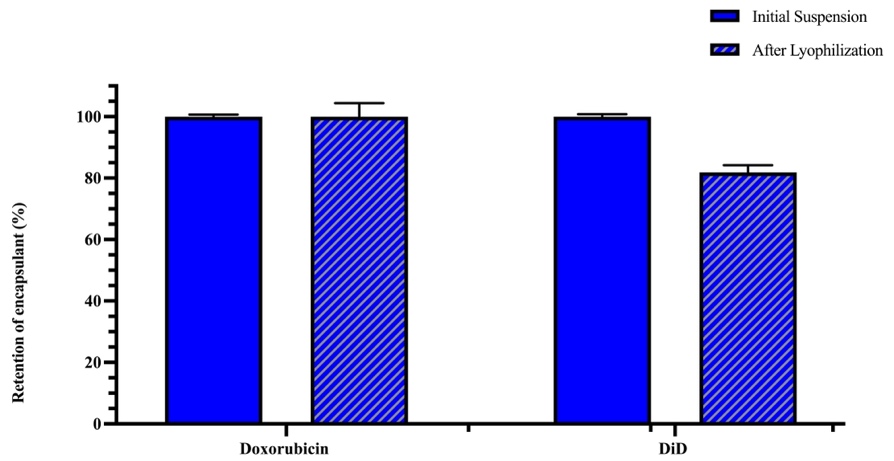 |
| **Figure S7** (a) Ac-Dex TPGS nanoparticles did not have a significant change in size after exposure to a 10% fetal bovine serum (FBS). (b) Nanoparticles achieved a 77% release of DiD after 72 hours of incubation in 10% FBS. The statistics were evaluated using an unpaired t-test. Data are reported as mean ± s.d. (n=3). |
| 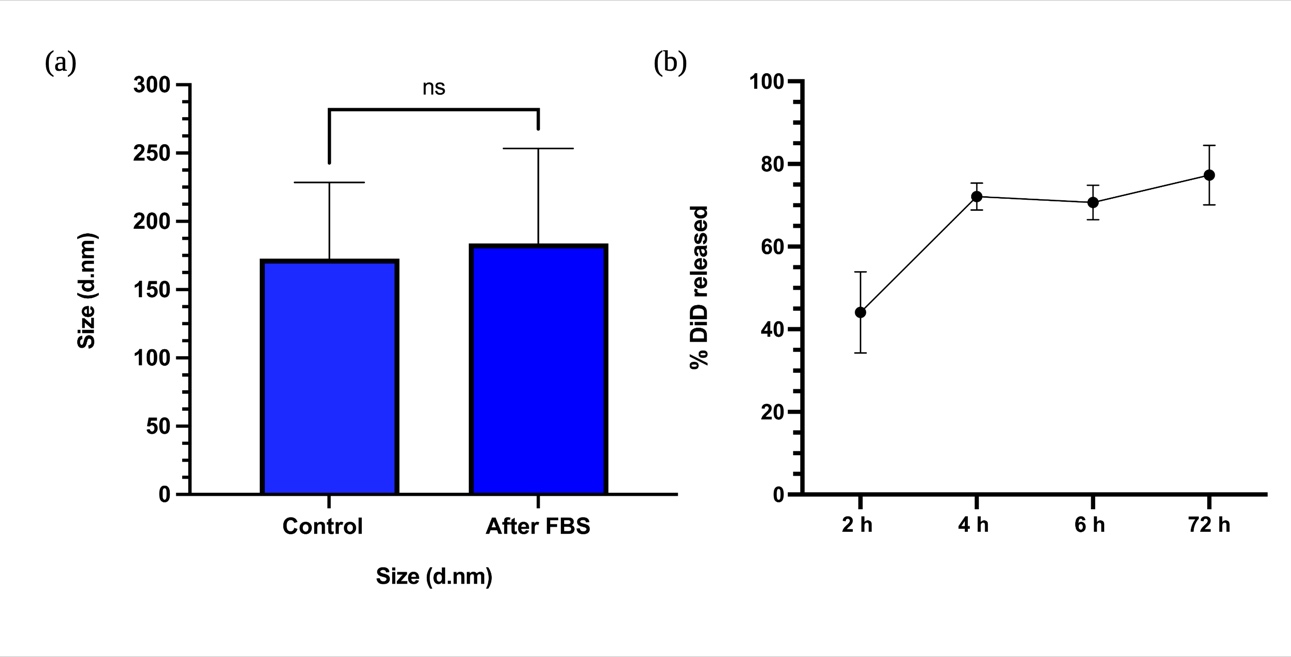 |

| **Figure S8** Ac-Dex F-127 nanoparticles were relatively nontoxic at all tested concentrations in Raw 264.7 macrophages at 48 hours. Data are reported as mean ± s.d. (n=4). |
| --- |
| 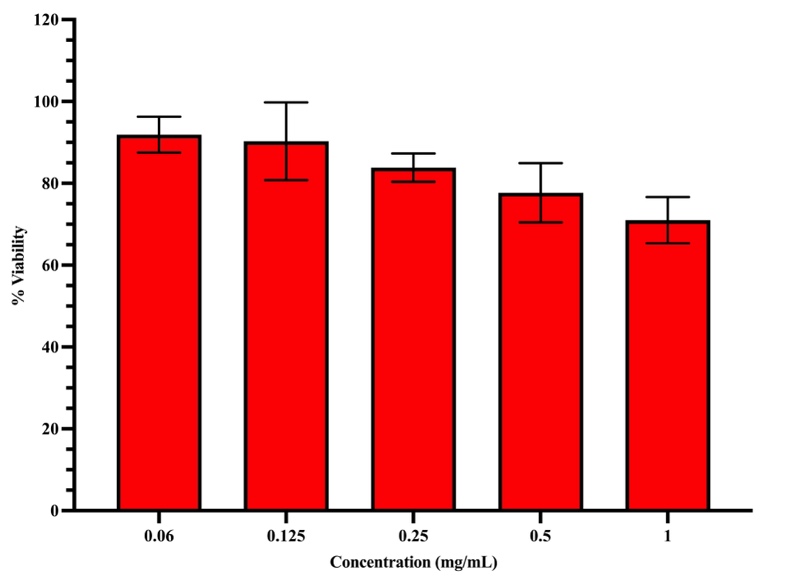 |

| **Figure S9** Ac-Dex TPGS nanoparticles show uptake that is maintained at 24 hours compared to what was achieved at 6 hours in Raw 264.7 macrophages. However, REH-CRL-8286 cells continue to show time-dependent uptake with increased cellular uptake at 24 hours. The statistics were evaluated using an unpaired t-test (*****p* < 0.0001). Data are reported as mean ± s.d. (n=3). |
| --- |
| 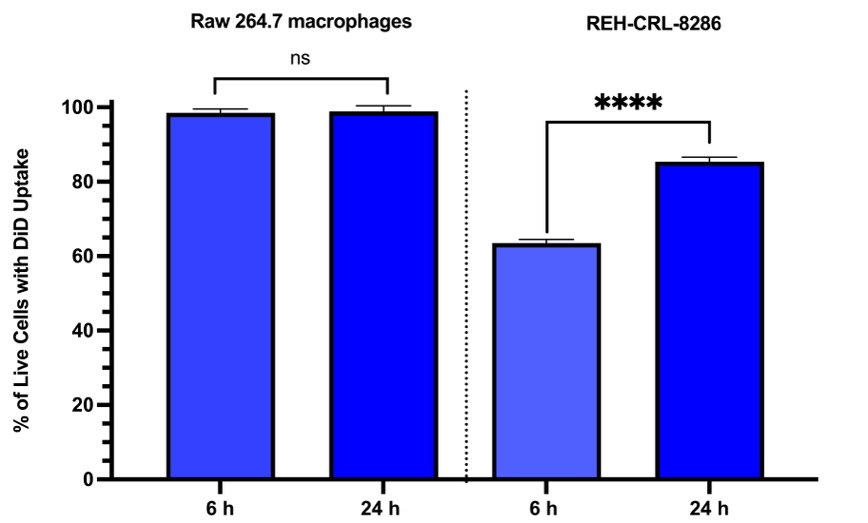 |

| **Figure S10** Ac-Dex F-127 nanoparticles show time-dependent endocytosis in Raw 264.7 macrophages, with uptake occurring within 15 minutes and increasing through 6 hours. Data are reported as mean ± s.d. (n=3). The statistics were evaluated using a one-way ANOVA with Tukey’s multiple comparison test. (** *p* < 0.01, *** *p* <0.0001) |
| --- |
| 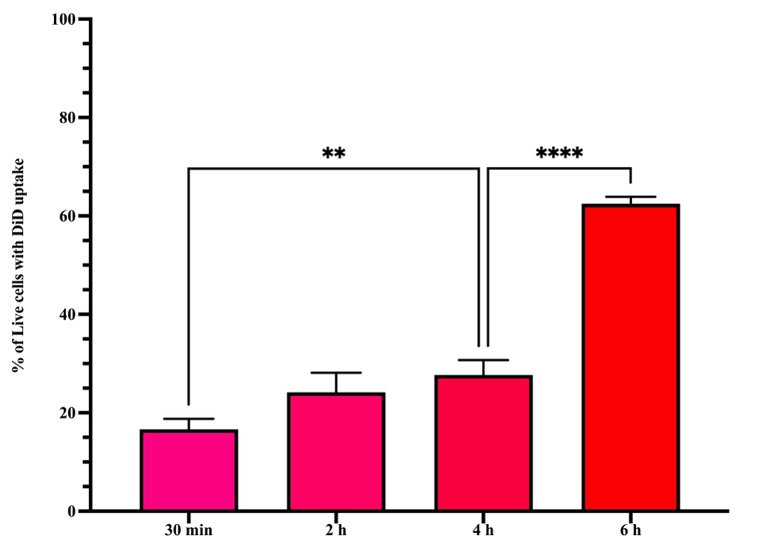 |

| **Figure S11** After the treatment with 15 µM chlorpromazine (CPZ) and 200 µM genistein endocytic inhibitors, there is a significant decrease in the cellular uptake of the nanoparticles at 6 hours for Ac-Dex formulations after inhibition with CPZ. Data are reported as mean ± s.d. (n=3). The statistics were evaluated using a one-way ANOVA with Tukey’s multiple comparison test. (** *p* <0.005). |
| --- |
| 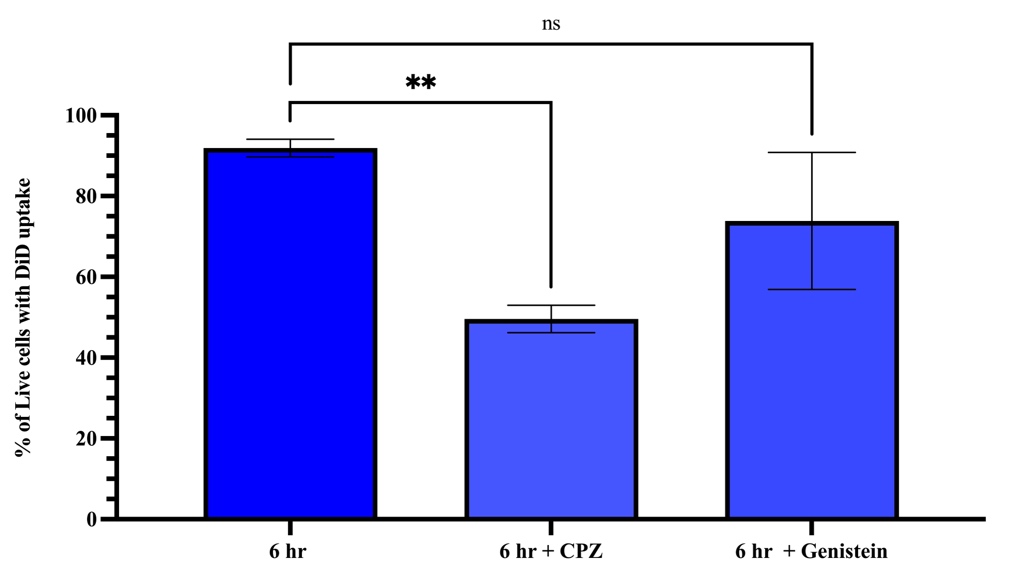 |

| **Figure S12** Ac-Dex F-127 nanoparticles release increasing amounts of payload when exposed to simulated endolysosomal conditions. The controlled release was determined in comparison to the release in PBS (pH of 7.4), representing the physiological state. (n=3). For 1-hour, nanoparticles were exposed to an environment that is pH 6.3, which is representative of the early endosome. After 4 hours, the nanoparticles were exposed to an environment with a pH of 5.3, which represents the late endosome. Finally, after 6 hours the nanoparticles were exposed to an environment with a pH of 4.3, which represents the endolysosome. The statistics were evaluated using a one-way ANOVA with Tukey’s multiple comparison test. (** *p* <0.005, (*** *p* <0.001, **** *p* <0.0001). Data are reported as mean ± s.d. (n=3).  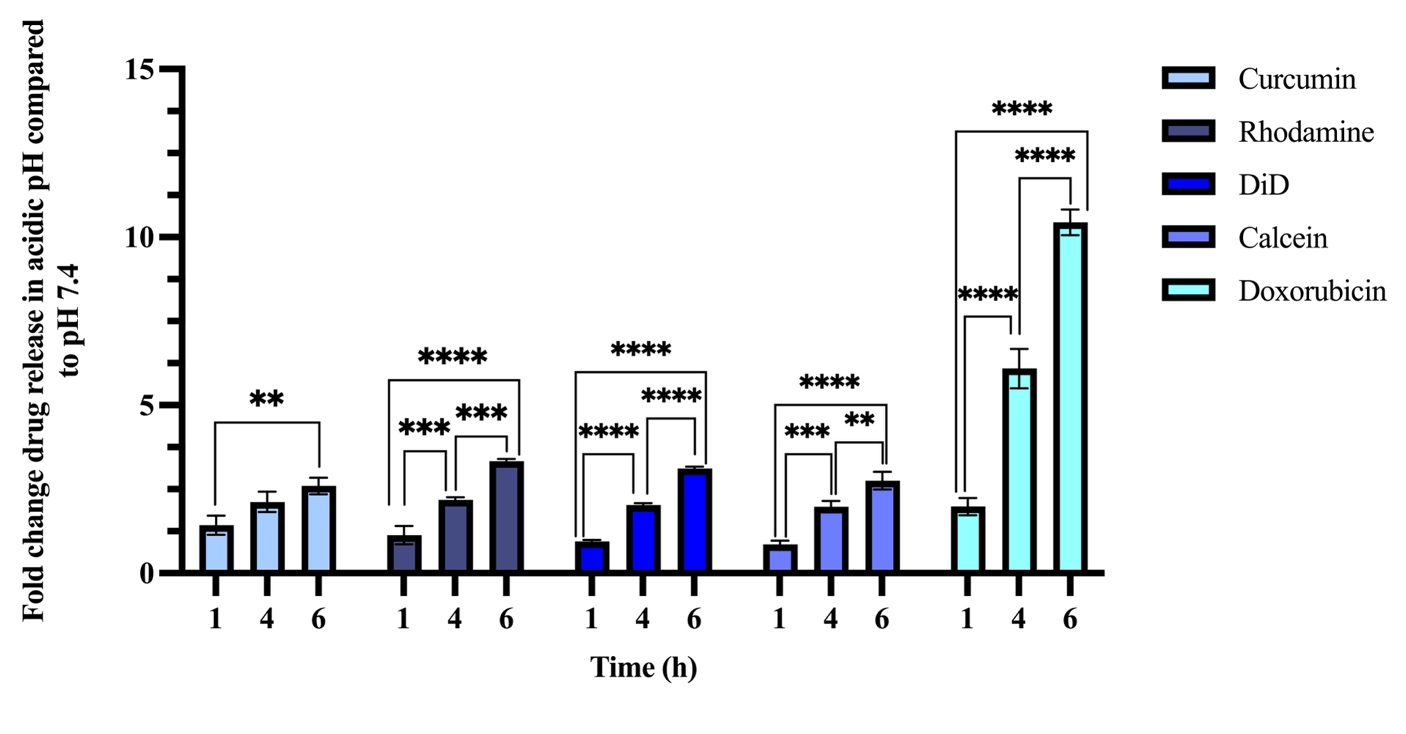 |
| --- |
| \| **Figure S13** Ac-Dex TPGS nanoparticles delivery and subsequent cytosolic release when loaded with rhodamine and incubated for 24 hours with Raw 264.7 macrophages. Each panel differentiates the nuclei (blue color), the rhodamine nanoparticles (red color), the brightfield showing the cell membrane, and the merged image of each channel overlay. Live cell images were taken with the 60X objective and cytosolic release is indicated inside the cell with a white arrow. The scale bar is 10 µm. \| \| --- \| \| 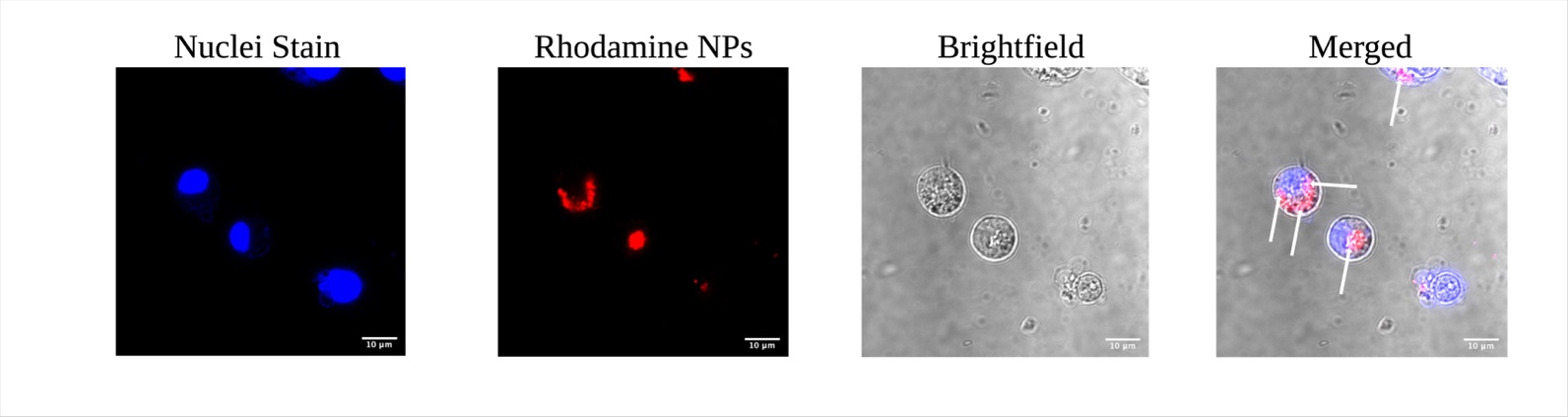 \|  \| \| **Figure S14** Ac-Dex F-127 nanoparticles delivery and subsequent cytosolic release when loaded with DiD and incubated for 24 hours with REH-CRL-8286 cells. Each panel differentiates the nuclei (blue color), the DiD nanoparticles (red color), the brightfield showing the cell membrane, and the merged image of each channel overlay. Live cell images were taken with the 60X objective and cytosolic release is indicated inside the cell with a white arrow. The scale bar is 10 µm. \| \| --- \| \| 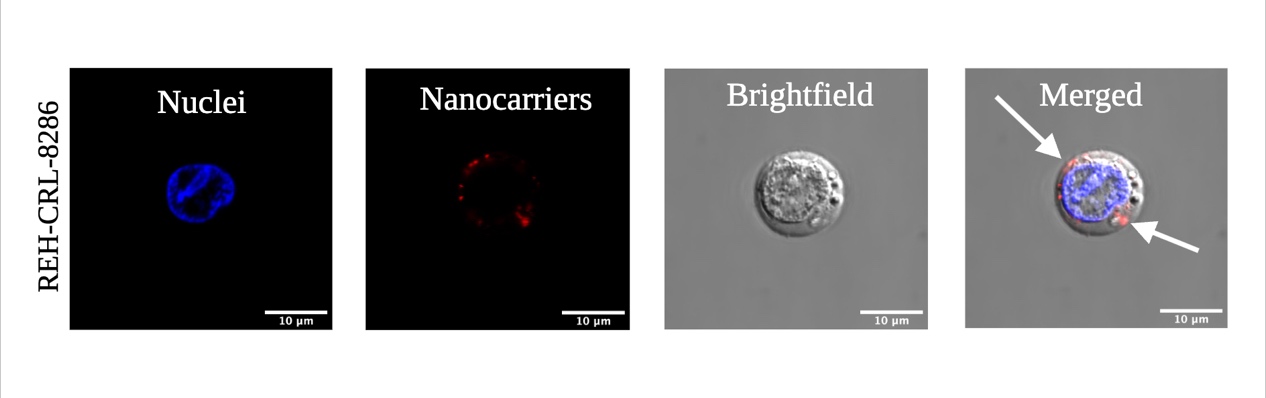 \| \| \| --- \| --- \| --- \| \|  \| |

|  |
| --- |
| Address correspondence to Sharan Bobbala, sharan.bobbala@hsc.wvu.edu |

**SI Reference**

[1] Kauffman, K. J.; Clement, D.; Sharma, S.; Gallovic, M. D.; Ainslie, K. M. **Synthesis and Characterization of Acetalated Dextran Polymer and Microparticles with Ethanol as a Degradation Product.** *J Am Chem Soc* **2012**, *4* (8), 4149–4155. https://doi.org/10.1021/am300888
